# Phospholipase C-related catalytically inactive protein (PRIP) controls KIF5B-mediated insulin secretion

**DOI:** 10.1242/bio.20147591

**Published:** 2014-05-08

**Authors:** Satoshi Asano, Tomomi Nemoto, Tomoya Kitayama, Kae Harada, Jun Zhang, Kana Harada, Isei Tanida, Masato Hirata, Takashi Kanematsu

**Affiliations:** 1 Department of Cellular and Molecular Pharmacology, Division of Basic Life Sciences, Institute of Biomedical and Health Sciences, Hiroshima University, Hiroshima 734-8553, Japan; 2 Laboratory of Molecular and Cellular Biophysics, Research Institute for Electronic Science, Hokkaido University, Sapporo 001-0020, Japan; 3 Laboratory of Biomembranes, Department of Biochemistry and Cell Biology, National Institute of Infectious Diseases, Tokyo 162-8640, Japan; 4 Laboratory of Molecular and Cellular Biochemistry, Faculty of Dental Science, Kyushu University, Fukuoka 812-8582, Japan

**Keywords:** GABARAP, Insulin secretion, KIF5B, PRIP, Vesicle transport

## Abstract

We previously reported that phospholipase C-related catalytically inactive protein (PRIP)-knockout mice exhibited hyperinsulinemia. Here, we investigated the role of PRIP in insulin granule exocytosis using *Prip*-knockdown mouse insulinoma (MIN6) cells. Insulin release from *Prip*-knockdown MIN6 cells was higher than that from control cells, and *Prip* knockdown facilitated movement of GFP-phogrin-labeled insulin secretory vesicles. Double-immunofluorescent staining and density step-gradient analyses showed that the KIF5B motor protein co-localized with insulin vesicles in *Prip*-knockdown MIN6 cells. Knockdown of GABA_A_-receptor-associated protein (GABARAP), a microtubule-associated PRIP-binding partner, by *Gabarap* silencing in MIN6 cells reduced the co-localization of insulin vesicles with KIF5B and the movement of vesicles, resulting in decreased insulin secretion. However, the co-localization of KIF5B with microtubules was not altered in *Prip*- and *Gabarap*-knockdown cells. The presence of unbound GABARAP, freed either by an interference peptide or by *Prip* silencing, in MIN6 cells enhanced the co-localization of insulin vesicles with microtubules and promoted vesicle mobility. Taken together, these data demonstrate that PRIP and GABARAP function in a complex to regulate KIF5B-mediated insulin secretion, providing new insights into insulin exocytic mechanisms.

## INTRODUCTION

Insulin secretion from pancreatic β-cells is a highly dynamic process regulated by multiple stimuli, including nutrients, hormones, and neuronal inputs. Stimulation by glucose induces a biphasic pattern of insulin release. First-phase insulin release occurs within a few minutes after exposure to elevated glucose, and it is followed by a more sustained second phase (Rorsman et al., 2000). The first phase, during which insulin granules that are predocked on the plasma membrane and/or recruited from a readily releasable pool become fused, has been extensively investigated. However, the precise mechanisms underlying the second phase are poorly understood. Second phase release correlates with mobilization of insulin-containing granules from the releasable pool into the cell periphery, which is mediated by microtubules. Insulin-containing cargo transport is regulated by the motor protein kinesin-1/KIF5 (Balczon et al., 1992; Cui et al., 2011; Meng et al., 1997; Varadi et al., 2002; Varadi et al., 2003).

Phospholipase C (PLC)-related but catalytically inactive protein (PRIP) was first identified as a novel inositol 1,4,5-trisphosphate-binding protein that has high homology to PLC-δ1, but lacks PLC activity (Kanematsu et al., 1996; Kanematsu et al., 1992; Kanematsu et al., 2000; Takeuchi et al., 1997; Takeuchi et al., 1996; Yoshida et al., 1994). We previously reported the effect of PRIP on inositol 1,4,5-trisphosphate-mediated Ca^2+^ release from the endoplasmic reticulum (Harada et al., 2005; Takeuchi et al., 2000). There are 2 isoforms of mammalian PRIP, PRIP1, which is present mainly in the brain, and PRIP2, which is ubiquitously expressed (Kanematsu et al., 1992; Kikuno et al., 1999; Uji et al., 2002). To elucidate the physiological function of PRIP, we identified a variety of PRIP-binding partners, including protein phosphatase 1α (Yoshimura et al., 2001), protein phosphatase 2A (Kanematsu et al., 2006; Sugiyama et al., 2012), GABA_A_ receptor-associated protein (GABARAP) (Kanematsu et al., 2002), the β subunits of GABA_A_ receptors (Terunuma et al., 2004), and the activated form of Akt (Fujii et al., 2010). These findings led us to investigate the possible involvement of PRIP in GABA_A_ receptor functions. PRIP regulates GABA_A_ receptor cell surface translocation and endocytosis through phosphoregulation of the receptor by these aforementioned PRIP-binding partners (Fujii et al., 2010; Kanematsu et al., 2007; Kanematsu et al., 2006; Mizokami et al., 2007; Terunuma et al., 2004). We also previously reported increased levels of plasma insulin in *Prip1*-knockout mice and serum gonadotropins in *Prip1* and *Prip2* double knockout (*Prip*-DKO) mice (Doira et al., 2001; Matsuda et al., 2009), suggesting an inhibitory role for PRIP in dense-core vesicle secretion.

GABARAP, a member of the microtubule-associated protein family, is composed of N-terminal (residues 1–22) and central (residues 40–68) regions that interact with microtubules and PRIP, respectively, and a C-terminal glycine at residue 116 that is covalently conjugated to phosphatidylethanolamine through a ubiquitination-like system (Ichimura et al., 2000; Kanematsu et al., 2005; Wang and Olsen, 2000). Northern blot analysis revealed that GABARAP expression is ubiquitous (Okazaki et al., 2000). Therefore, GABARAP can regulate a variety of cell functions, including the functions of GABA_A_ receptor-expressing neurons. Indeed, GABARAP has been reported to promote the cell surface expression of angiotensin II type I receptor (Cook et al., 2008), transient receptor potential vanilloid 1 (Laínez et al., 2010), and κ-opioid receptor (Chen et al., 2011) by facilitating their trafficking along the secretory vesicle pathway. GABARAP interacts with PRIP and regulates the recruitment of γ subunit-containing GABA_A_ receptors to the cell surface (Boileau et al., 2005; Chen et al., 2005; Kanematsu et al., 2002; Leil et al., 2004; Mizokami et al., 2007).

In this study, we knocked down *Prip* or *Gabarap* in the MIN6 mouse insulinoma cell line using specific small interfering RNAs (siRNAs), and then analyzed glucose-induced insulin secretion from these cells. In addition, we investigated the importance of PRIP and GABARAP interaction in insulin vesicle movement. Finally, by analyzing pancreatic islets from wild-type and *Prip*-knockout mice, we proposed that PRIP regulates second phase insulin secretion.

## MATERIALS AND METHODS

### Antibodies and reagents

An anti-GABARAP antibody (PM037) that recognizes the N-terminal region (amino acids 1–39) was from Medical and Biological Laboratories (Nagoya, Japan). Anti-insulin/proinsulin antibody (2IP10) was from HyTest (Turku, Finland). Anti-KIF5 antibody (SAB3500282), anti-β-tubulin antibody (D66), and anti-SNAP25 antibody (S5187) were from Sigma–Aldrich (St Louis, MO, USA). Anti-Ptprn2 (phogrin) antibody (PAB15812) was from Abnova (Taipei, Taiwan). Anti-Rab27a antibody (17817-1-AP) was from Proteintech (Chicago, IL, USA). Anti-HaloTag antibody was from Promega (Madison, WI, USA). Anti-tubulin beta antibody (RB-9249) was from Thermo Fisher Scientific (Waltham, MA, USA). Anti-syntaxin1 antibody (sc-12736) was from Santa Cruz Biotechnology (Santa Cruz, CA, USA). Anti-PRIP1 and anti-PRIP2 polyclonal antibodies were described previously (Kanematsu et al., 2002; Otsuki et al., 1999). FITC-conjugated anti-myc antibody (R953-25), Alexa Fluor 488 anti-rabbit IgG (A11008), and Alexa Fluor 555 and 405 anti-mouse IgG (A21422 and A31553, respectively) antibodies were from Invitrogen (Carlsbad, CA, USA). Peroxidase-conjugated anti-rabbit (P0399) and anti-mouse (NA9310) IgG antibodies were from Dako (Glostrup, Denmark) and GE Healthcare (Pittsburgh, PA, USA), respectively. TurboGFP-C-terminally tagged mouse Ptprn2/phogrin construct (NM_011215) was from OriGene (Rockville, MD, USA). *Gabarap*-siRNA (1, 2) (s80666, s80665) and *Kif5b*-siRNA (1, 2) (s68783, s68781) were from Life Technologies (Carlsbad, CA, USA). Mouse *Prip1*-siRNAs (1, 2, 3) and *Prip2*-siRNAs (1, 2, 3) were described previously (Kitayama et al., 2013).

### Plasmid construction

YFP-tagged human GABARAP (Tanida et al., 2006) was kindly provided by Dr E. Kominami (Juntendo University, Japan). GABARAP40–67 was cloned into the pIRES2-DsRed-Express vector (Takara, Shiga, Japan) and a HaloTag-containing pcDNA3.1 vector (produced in our laboratory). GABARAP2-35 was cloned as a fusion with a myc epitope. DsRed-PRIP1 has been described previously (Kanematsu et al., 2006).

### Cell culture and transfection

The MIN6 cell line, obtained with permission from Dr Jun-ichi Miyazaki at Osaka University (Miyazaki et al., 1990), was cultured in high glucose Dulbecco's Modified Eagle's medium (DMEM; Sigma–Aldrich) containing 15% fetal bovine serum (FBS) at 37°C and 5% CO_2_. Cells were transfected with plasmid constructs or siRNAs using Lipofectamine 2000 (Invitrogen), and after incubation for 24–48 h, experiments were performed as described below.

### Insulin secretion assay

For the insulin secretion assay, 30 isolated pancreatic islets placed in a μ-Slide I^0.8^ chamber (Ibidi, Martinsried, Germany) or subconfluent MIN6 cells seeded in 12-well culture plates were pre-incubated in Krebs–Ringer buffer (119 mM NaCl, 4.74 mM KCl, 1.19 mM MgCl_2_, 2.54 mM CaCl_2_, 1.19 mM KH_2_PO_4_, 25 mM NaHCO_3_, 10 mM HEPES-NaOH [pH 7.4], and 0.5% BSA) containing 2.8 mM glucose (for islets) or 5 mM glucose (for MIN6 cells) for 30 min at 37°C, and then stimulated with Krebs–Ringer buffer containing high concentrations of glucose for the indicated times. Aliquots of the high-glucose stimulation medium (0.2 or 0.5 mL, respectively) were collected every 1 min, and then the same amount of fresh medium was added. To determine insulin content, islets and MIN6 cells were lysed in 1% Triton X-100/phosphate-buffered saline (PBS) for 30 min at 4°C. Insulin concentration was measured using a mouse insulin ELISA kit (Mercodia, Uppsala, Sweden) according to the manufacturer's instructions.

### Fractionation by discontinuous OptiPrep™ step-gradient

Membrane fractionation was performed using an OptiPrep™ gradient (AXIS-Shield, Oslo, Norway). Briefly, cells were harvested and suspended in homogenization buffer (0.25 M sucrose, 20 mM KCl, 1 mM EDTA, 20 mM HEPES-KOH [pH 7.6], and a mixture of protease inhibitors), and then lysed by passage (20 times) through a fine-gauge syringe needle. The homogenates were centrifuged at 1,000 × *g* for 10 min to obtain a postnuclear supernatant. The cell lysates were loaded onto an OptiPrep™ gradient consisting of 3%, 7.5%, 18%, and 35% (w/v) iodixanol solutions. Centrifugation was performed in a Beckman SW 41 rotor at 100,000 × *g* for 16 h at 4°C. Eighteen fractions were collected from the top of each centrifuge tube and analyzed by SDS-PAGE (or a dot blot for insulin detection) followed by western blotting.

### Immunofluorescence

Cells on coverslips were fixed with 3.7% paraformaldehyde/PBS for 30 min. Cells were permeabilized with 0.2% Triton X-100 for 4 min, and then incubated with 1% bovine serum albumin/PBS for 15 min. The cells were incubated with the primary antibody for 1 h, and then incubated with the Alexa Fluor-conjugated secondary antibody for 1 h. Subsequently, the cells were mounted on a microscope slide with Perma Fluor Aqueous Mounting Medium (Thermo Fisher Scientific) and observed with a confocal laser scanning microscope (Fluoview FV10i; Olympus) using a 60×/1.35 NA oil-immersion lens. Images were acquired using FV 10i SW software (Olympus).

### Live imaging of secretory vesicles and image analysis

Cells transfected with GFP-phogrin were stimulated with 30 mM glucose in Krebs–Ringer buffer. The movement of GFP-phogrin vesicles was observed every 5 sec over a 90-sec period by live-cell imaging in a cell observation chamber mounted on a Biozero fluorescent microscope (BZ-9000; Keyence, Osaka, Japan) with a Plan Apo 100×/1.40 NA oil lens (Nikon). Images were acquired and deconvoluted using BZ-II Viewer software (Keyence). Tracking analysis of secretory granules in the images was performed using the manual tracking plug-in (Fabrice Cordelieres, Orsay, France) and the chemotaxis and migration tool (Ibidi) of ImageJ 1.43u (National Institutes of Health, Bethesda, MD, USA) as described in the μ-Slide Chemotaxis protocol.

### Co-localization analysis

Co-localization was assessed with the WCIF ImageJ intensity correlation analysis plug-in (developed by W. Rasband). Each image that showed 2 proteins was analyzed with 4 images. Specifically, 2 of these images showed the co-localization of the 2 proteins after background removal for both the red and green channels. The third image was an overlay of the red and green signals. The fourth is a pseudo-color PDM [PDM for each pixel = (red intensity−mean red intensity)×(green intensity−mean green intensity)] image in which the areas of high and low co-localization are shown in yellow and blue, respectively, and the areas without correlation between the signals for the 2 proteins are shown in black. The plug-in also provided the *R*r value (ranging between −1 and 1, where 1 is a perfect correlation), the *R* value (ranging between 1 and 0, where 1 is high co-localization and 0 is low co-localization), the *M*1 and *M*2 values, and a co-localization PDM image in which the blue and yellow areas correspond to the absence and presence of co-localization, respectively.

### Immunoprecipitation assay

The association of GABARAP with PRIP was analyzed using MIN6 cells expressing myc-tagged GABARAP with or without high glucose stimulation. Cells were homogenized in a buffer containing 20 mM HEPES-Na (pH 7.5), 150 mM NaCl, 0.1% Triton X-100, 10% (w/v) glycerol, and a mixture of protease inhibitors, and then centrifuged at 13,000 × *g* for 60 min at 4°C. The resulting supernatant was incubated with 5 µg of anti-myc antibody or control rabbit IgG, and then incubated with protein G-Sepharose overnight at 4°C with gentle rotation. After gentle centrifugation, the precipitates were boiled in SDS sample buffer, separated by SDS-PAGE, and analyzed by western blotting using anti-PRIP1 and anti-PRIP2 antibodies. To visualize the antibody–protein complexes, SuperSignal West Femto extended duration substrate (Thermo Fisher Scientific) was used. Imaging of western blots was performed using an ImageQuant LAS 4000 mini (GE Healthcare).

### Isolation of mouse pancreatic islets

Experimental procedures involving animals and animal handling were performed according to the guidelines of Hiroshima University and were approved by the Animal Care and Use Committee of Hiroshima University. Eight- to twelve-week-old *Prip*-DKO mice (Mizokami et al., 2007) were killed by cervical dislocation, and pancreatic islets were isolated by collagenase digestion (Takahashi et al., 1997). Islets were maintained for 12 h in DMEM containing 10% FBS at 37°C and 5% CO_2_.

### Two-photon excitation imaging

Exocytosis was visualized by using a fluid-phase tracer, sulforhodamine B (0.7 mM; Molecular Probes, Carlsbad, CA, USA) in an assay solution (140 mM NaCl, 5 mM KCl, 2 mM CaCl_2_, 1 mM MgCl_2_, and 10 mM HEPES-NaOH) containing 2.8 mM (low) or 20 mM (high) glucose with two-photon excitation imaging. The islets were imaged with an inverted microscope (IX70; Olympus, Tokyo, Japan) and a laser-scanning microscope (FluoView; Olympus) equipped with a water-immersion objective lens (UplanApo60xW/IR; NA, 1.2), as previously described (Kasai et al., 2005). Two-photon excitation was performed at 830 nm, and the fluorescence signals of the Ca^2+^ indicator fura-2 (Kd: ∼200 nM) and the polar tracer sulforhodamine B were separated by a dichroic filter and captured at 420–560 and 570–650 nm, respectively, and images were acquired every 1 sec. In an individual, 4,500–5,500 µm^2^ region of interest in islets containing approximately 40–60 cells, we analyzed abruptly appearing small fluorescent spots on the plasma membrane, which were recorded as “exocytic events”.

### Statistical analysis

Data are presented as mean ± s.e. or s.d. Original data were compared by the Kruskal–Wallis test, and further comparisons between 2 conditions were performed by Dunn's multiple comparison test, unless otherwise stated.

## RESULTS

### PRIP gene silencing in MIN6 cells increases insulin secretion and the mobility of insulin vesicles

**Fig. 1.**
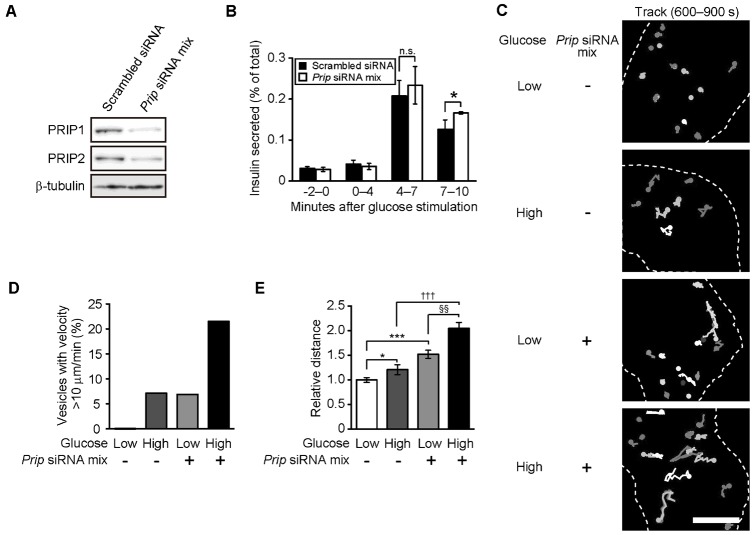
Silencing of *Prip1* and *Prip2* in MIN6 cells promotes insulin secretion and vesicle movement. (A) *Prip* knockdown in MIN6 cells. MIN6 cells were transfected with mixture of *Prip* siRNAs [*Prip* siRNA mix; *Prip1*-siRNAs (1, 2, 3) and *Prip2*-siRNAs (1, 2, 3)], cultured for 2 days, and then lysed. The whole cell lysates were processed for western blotting. Equivalent amounts of protein were loaded into each well (see β-tubulin staining). We obtained similar results from 3 different experiments, and a set of typical images is shown. (B) Enhanced insulin release from *Prip*-knockdown MIN6 cells. *Prip*-siRNA mix-transfected MIN6 cells were stimulated with 30 mM glucose, and the released insulin was measured every 1 min. Insulin secretion was normalized to intracellular insulin content, and is presented as a percentage of the total intracellular content. Values are presented as mean ± s.d. (*n* = 4). (C) Track plot recording of insulin vesicles in *Prip*-knockdown MIN6 cells. Cells were maintained in 5 mM glucose or stimulated with 30 mM glucose for 600 sec, and a time-lapse series of GFP-phogrin images was obtained every 5 sec for up to 690 sec. Particles of GFP-phogrin were selected at random, and each track, shown as a white line, was plotted based on the images. The dotted line shows a cell edge. We repeated these experiments 3 times, and analyzed approximately 70 displaced vesicles. A representative set of images is shown. (D) Analysis of vesicle mobility using GFP-phogrin-labeled vesicles. The data from 3 experiments were combined, and the percentage of vesicles travelling at a velocity greater than 10 µm per min is shown. (E) The average accumulated distance per vesicle was calculated. Each relative value is based on the distance traveled by control cells (without *Prip*-siRNA transfection) under low-glucose conditions. Values are presented as mean ± s.e. (from the left in each indicated condition; *n* = 70, 78, 81, and 63, respectively). *p<0.05, ^§§^*p*<0.01, ***p<0.001, ^†††^*p*<0.001; n.s., not statistically significant. Scale bar: 5 µm.

*Prip1*-knockout mice exhibit a hyperinsulinemic phenotype (Doira et al., 2001). To verify the involvement of PRIP in the regulation of insulin granule exocytosis, we performed an insulin secretion assay using MIN6 cells transfected with both *Prip1*-siRNAs (1, 2, 3) and *Prip2*-siRNAs (1, 2, 3) as well as a scrambled control siRNA. The expression of PRIP1 and PRIP2 was assessed by western blotting, which showed that their expression was markedly reduced within 2 days after transfection (Fig. 1A). To verify this effect, a different pair of *Prip1*- and *Prip2*-siRNAs was transfected into MIN6 cells, and these siRNAs also reduced PRIP1 and PRIP2 expression (supplementary material Fig. S1A). The release of insulin was then measured before and after stimulation with high glucose (30 mM). Samples were successively collected at 0–4 min, 4–7 min, and 7–10 min after stimulation. In control cells, insulin release was 6.8-fold and 4.3-fold higher than the basal level (before stimulation, −2–0 min) at 4–7 and 7–10 min, respectively (Fig. 1B). Similar 7.0-fold and 5.4-fold increases were also observed in *Prip*-knockdown cells during the same time intervals. The upregulation of insulin was significant in the *Prip*-knockdown cells at 7–10 min, suggesting that PRIP negatively regulates insulin release in MIN6 cells, particularly at the late phase. A similar result was observed when another pair of *Prip* siRNAs [*Prip1*-siRNA (3) and *Prip2*-siRNA (3)] was used (supplementary material Fig. S1D).

Insulin vesicles are transported from the releasable pool to the cell surface, and then secreted (Rorsman et al., 2000). PRIP regulates neuronal surface expression of γ subunit-containing GABA_A_ receptors by controlling GABARAP function (Mizokami et al., 2007). These findings suggest that PRIP and/or GABARAP may participate in the transport of insulin vesicles. To determine if PRIP participates in insulin vesicle movement, we performed time lapse imaging using GFP-tagged phogrin, which has been reported to localize to the membrane of insulin secretory granules (Wasmeier and Hutton, 1996), allowing for monitoring of anterograde insulin-containing vesicle translocation to the cell surface (Varadi et al., 2002). First, we evaluated the localization of phogrin and insulin vesicles in MIN6 cells by immunocytochemistry (supplementary material Fig. S2). Both signals were highly co-localized, indicating that insulin vesicle movement can be monitored by following GFP-tagged phogrin movement.

Transfection of *Prip*-siRNAs into MIN6 cells appeared to trigger the vigorous movement of insulin vesicles, even in low glucose conditions, which was quantitatively analyzed by measuring the rate of moving vesicles, and vigorous movement was defined as vesicles moving with a velocity exceeding 10 µm per min (Nakajima et al., 2012). The mean mobility index was also increased in *Prip*-knockdown cells (Fig. 1C–E). When the cells were stimulated with high glucose, the percentage of vigorously moving vesicles during the period from 600 sec to 690 sec after stimulation was increased in *Prip*-knockdown cells (Fig. 1C,D), and the accumulated distance was approximately 2-fold higher (Fig. 1E). These results suggest that PRIP deficiency promotes phogrin-positive vesicle movement.

### KIF5B-regulated insulin vesicle transport in MIN6 cells is enhanced by *Prip* silencing

**Fig. 2.**
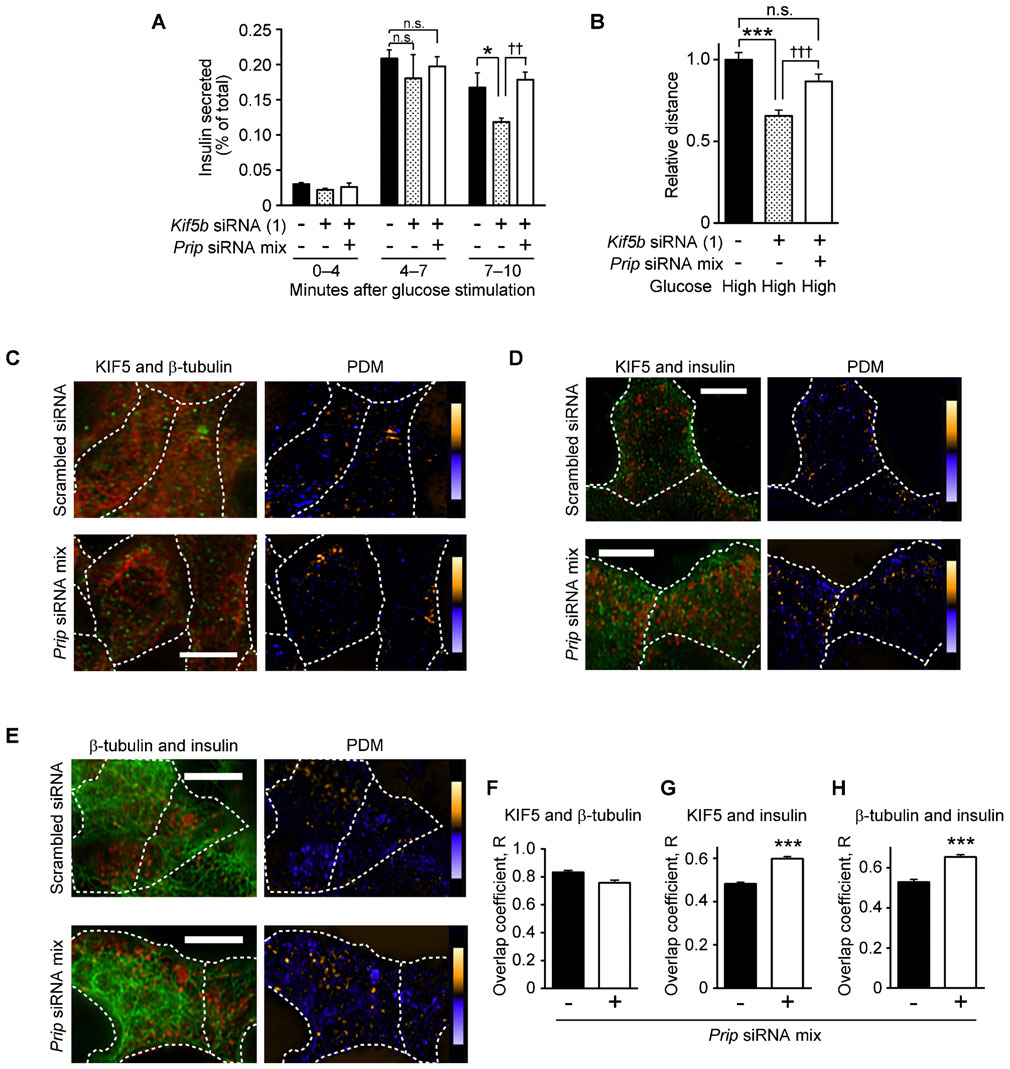
KIF5B-mediated insulin vesicle transport is regulated by PRIP. (A) Insulin secretion analysis of *kif5b*-knockdown MIN6 cells. MIN6 cells transfected with *Kif5b*-siRNA (1) and *Prip*-siRNA mix (+) or scrambled siRNA (−) were stimulated with 30 mM glucose. Released insulin was collected and measured every 1 min. The mean percentage of total insulin content is shown. Values are presented as mean ± s.d. (*n* = 3). (B) The average accumulated distance per vesicle was calculated. Each value is the average distance travelled by the cells relative to that of cells transfected with scrambled siRNA under high glucose (30 mM) conditions. Values are presented as mean ± s.e. (from the left in each experiment; *n* = 120, 116, and 56, respectively). (C–E) Co-localization analyses of KIF5 (green) and β-tubulin (red) (C), KIF5 (green) and insulin (red) (D), and β-tubulin (green) and insulin (red) (E) in MIN6 cells transfected with scrambled siRNA (upper panels) or *PRIP1*-siRNAs (1, 2, 3) and *PRIP2*-siRNAs (1, 2, 3) (*Prip*-siRNA mix; lower panels). Cells were stimulated with 30 mM glucose for 10 min, fixed with 3.7% paraformaldehyde, subjected to immunocytochemistry with a specific antibody, and processed for confocal microscopy. The yellow and blue pseudo-colors in the PDM images show areas of high and low co-localization, respectively. Magnified images of panels C–E are shown in supplementary material Fig. S8A–C. The dotted line shows a cell edge. A set of typical images from 3 independent experiments is shown. (F–H) Statistical analysis of the co-localization experiments in panels C–E. Overlap coefficients were calculated in each experiment. Values are presented as mean ± s.d. (from the left in each graph, *n* = 60 and 45 (F); 96 and 104 (G); 84 and 60 (H), respectively). *p<0.05, ***p<0.001, ^††^*p*<0.01, ^†††^*p*<0.001; n.s., not statistically significant. Scale bars: 5 µm.

Insulin vesicle movement is regulated by kinesin-1 (conventional kinesin). KIF5, the heavy chain of kinesin-1, has three subtypes, KIF5A, KIF5B, and KIF5C; KIF5B is only detected in isolated mouse pancreatic islets and participates in insulin secretion (Cui et al., 2011). By using reverse transcription-polymerase chain reaction, we confirmed that MIN6 cells expressed *Kif5b*, but not *Kif5a* or *Kif5c* (supplementary material Fig. S3A). To examine the involvement of KIF5B in insulin secretion, we analyzed insulin secretion and insulin vesicle mobility in MIN6 cells transfected with two different *Kif5b*-siRNAs (1 and 2). KIF5B expression, as assessed by western blotting, was markedly reduced but not completely abolished (supplementary material Fig. S1B). KIF5B signals were rarely observed in *Kif5b*-siRNA (1)-transfected cells by immunocytochemical analysis (supplementary material Fig. S3B). Insulin secretion was assayed by measuring insulin levels in culture medium collected at 0–4 min, 4–7 min, and 7–10 min after high glucose stimulation (Fig. 2A). The upregulation of insulin secretion at 7–10 min following high glucose stimulation was significantly decreased in *Kif5b*-siRNA (1) knockdown cells, whereas insulin secretion was rebounded when *Prip1*, *Prip2*, and *Kif5b* were simultaneously knocked down (Fig. 2A). Similar patterns were also observed in an insulin vesicle mobility assay (Fig. 2B). Presumably, the rebound observed following simultaneous *Prip* and *Kif5b* knockdown was the result of the enhanced function of the remaining endogenous KIF5B, suggesting an inhibitory role for PRIP in kinesin-mediated insulin secretion. A similar result was obtained in cells transfected with a different *Kif5b*-siRNA (2) in an insulin secretion assay (supplementary material Fig. S1D).

We then examined the relationship between PRIP and KIF5B in insulin vesicle transport under high glucose conditions. Co-localization analysis of KIF5, β-tubulin (microtubules), and insulin vesicles was performed by double-staining immunocytochemistry in *Prip*-siRNA-transfected MIN6 cells. The localization of KIF5 and β-tubulin in scrambled siRNA-transfected and *Prip*-knockdown cells were not different (representative images and the overall data are shown in Fig. 2C and Fig. 2F, respectively). However, the co-localization of KIF5 with insulin vesicles and of insulin vesicles with β-tubulin was significantly enhanced (for KIF5 and β-tubulin, see Fig. 2D,G and Fig. 2E,H, respectively). These data suggest that PRIP deficiency promotes the interaction of insulin vesicles with KIF5 without affecting the co-localization between KIF5 and microtubules.

**Fig. 3.**
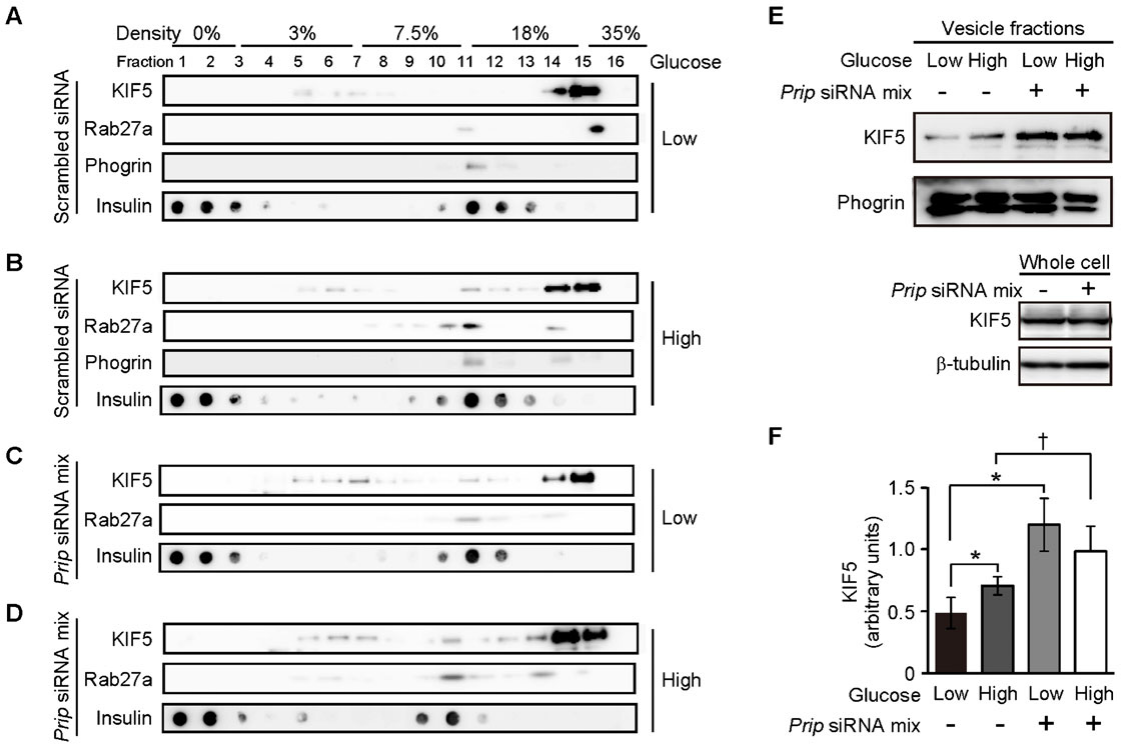
Knockdown of *Prip* increases the amount of KIF5 in the secretory vesicle fraction. (A–D) Cells were transfected with scrambled siRNA (A,B) or both *Prip1*-siRNAs (1, 2, 3) and *Prip2*-siRNAs (1, 2, 3) (*Prip* siRNA mix; C,D). The cells were stimulated with (B,D) or without (A,C) 30 mM glucose for 10 min. Equivalent protein in the cell homogenates was fractionated in OptiPrep™ discontinuous gradients (3%, 7.5%, 18%, and 35%). The fractions were analyzed by western blotting using the indicated specific antibodies. We performed each experiment 3 times, and a set of typical images is shown. (E,F) The vesicle fraction (fraction 11), which was defined as the fraction with high levels of Rab27a, phogrin, and insulin, was collected, and KIF5 content was analyzed by western blotting. The total amounts of KIF5 in *Prip*-knockdown (+) and scrambled siRNA-transfected cells (−) did not differ (lowest blot in panel E). A set of typical images is shown. The calculated mean value for each band is shown in the graph (F). Values are presented as mean ± s.d. (*n* = 3); *p<0.05, ^†^*p*<0.05.

To further analyze the distribution of insulin vesicles with KIF5 in MIN6 cells, we carried out an OptiPrep™ discontinuous gradient fractionation assay. The secretory granule proteins phogrin and Rab27a (Arden et al., 2004; Yi et al., 2002) were located in fraction 11, which was at the interface between 7.5% and 18% OptiPrep™ where insulin was also accumulated (Fig. 3A,B). Therefore, fraction 11 was designated the insulin dense-core granular fraction. In response to high glucose stimulation, KIF5 was detected in fraction 11 (compare Fig. 3A to Fig. 3B). *Prip* deficiency appeared to induce KIF5 localization with the secretory vesicle fraction under low glucose conditions, and this was unchanged when cells were stimulated with high glucose (compare Fig. 3A to Fig. 3C and Fig. 3D). The increased KIF5 in the secretory vesicle fraction was then quantified by western blotting (Fig. 3E,F). *Prip* deficiency caused a 2.4-fold increase in the amount of KIF5 present in the vesicle fraction compared to the amount in the control MIN6 cells under low glucose conditions, and the increase was unchanged under high glucose conditions (Fig. 3F), indicating that *Prip* deficiency promotes the co-localization of insulin vesicles with KIF5B, independent of extracellular glucose concentration.

### GABARAP and PRIP are required for insulin secretion

**Fig. 4.**
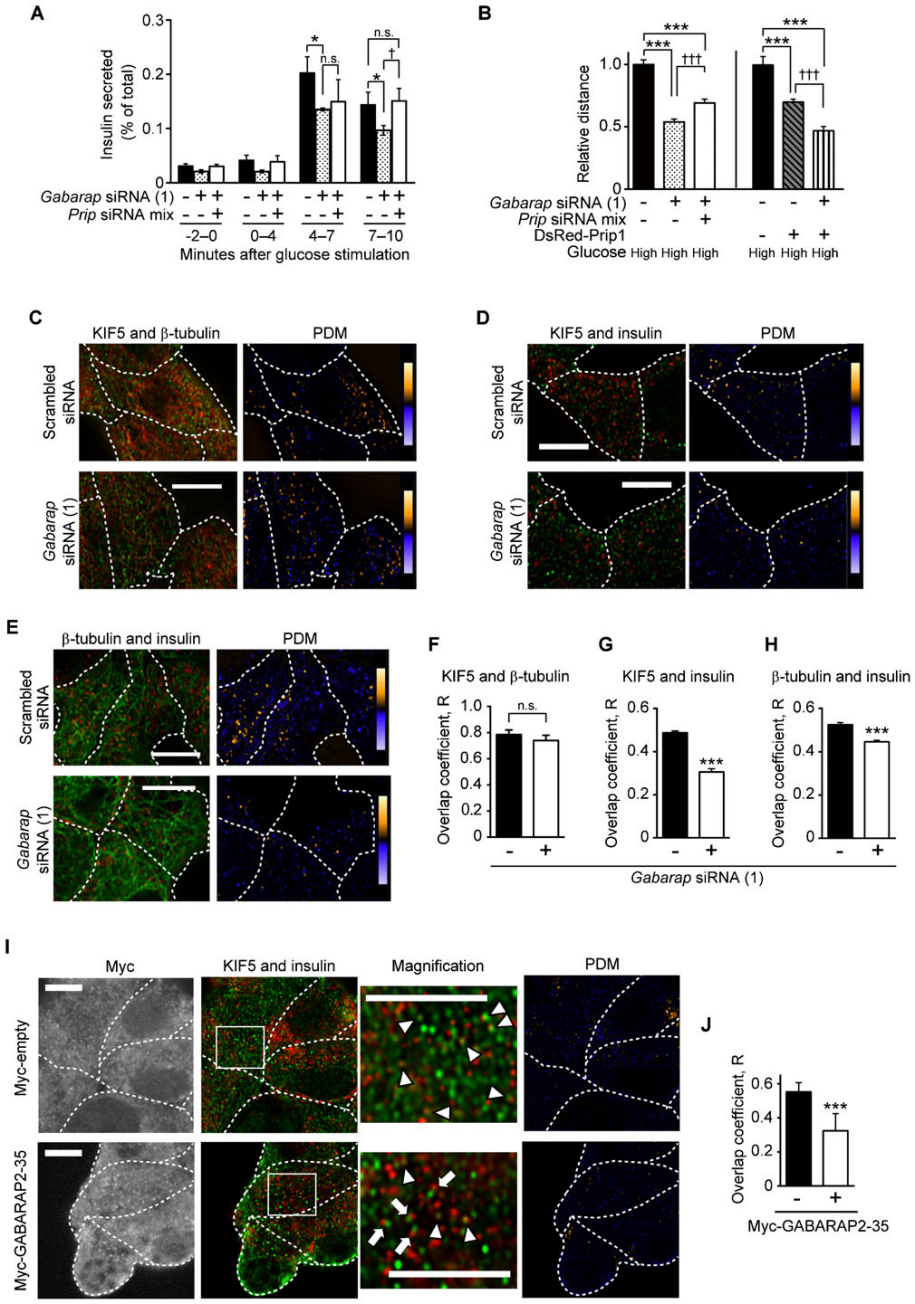
GABARAP enhances insulin vesicle transport by regulating insulin vesicle localization with KIF5 and β-tubulin, which is negatively regulated by PRIP. (A) Insulin secretion from *Gabarap*-knockdown MIN6 cells. *Gabarap*-siRNA-transfected MIN6 cells co-transfected with or without *Prip1*-siRNAs (1, 2, 3) and *Prip2*-siRNAs (1, 2, 3) (*Prip* siRNA mix) were cultured for 2 days. The cells were stimulated with 30 mM glucose, and the released insulin was measured every 1 min. The mean value for the percentage of total insulin content is shown. Values are presented as mean ± s.d. (*n* = 3). (B) The mean accumulated distance per vesicle was calculated under high-glucose conditions. Each value is presented relative to the distance traveled by the control (black bar). Values are presented as mean ± s.e. (from the left, *n* = 47, 168, 112, 45, 169, and 66). (C–J) Co-localization analyses of KIF5 (green) and β-tubulin (red) (C), KIF5 (green) and insulin (red) (D), and β-tubulin (green) and insulin (red) (E) in MIN6 cells transfected with scrambled siRNA (upper panels) or *Gabarap*-siRNA (1) (lower panels), or KIF5 and insulin (I) in MIN6 cells transfected with an empty vector (upper panels) or myc-tagged GABARAP2–35 (lower panels) after 30 mM glucose stimulation for 10 min. Cells were fixed with 3.7% paraformaldehyde, subjected to immunocytochemistry with each specific antibody, and processed for confocal microscopy. The yellow and blue pseudo-colors in the PDM images show areas of high and low co-localization, respectively. Magnified images of panels C–E are shown in supplementary material Fig. S8A–C. The myc-expressing cells (the left panel of each experiment) isolated with a FITC-conjugated anti-myc antibody were analyzed (I), and the arrowheads and arrows in the magnified images represent the areas of co-localization and non-colocalization between KIF5 (obtained blue images were replaced with green) and insulin (red), respectively. The single-color images are shown in supplementary material Fig. S9A. The dotted line shows a cell edge. A set of typical images from more than 81 cells in 3 independent experiments is shown. The overlap coefficient was calculated and is shown in panels F–H and J, and the values are presented as mean ± s.d. (from the left in each graph, *n* = 96 and 100; 128 and 124; 124 and 100; 93 and 81, respectively). *p<0.05, ***p<0.001, ^†^*p*<0.05, ^†††^*p*<0.001; n.s., not statistically significant. Scale bars: 5 µm.

PRIP binds directly to GABARAP, and this complex facilitates the recruitment of GABA_A_ receptors to the cell membrane (Mizokami et al., 2007). GABARAP is a tubulin-binding protein that regulates the trafficking of GABA_A_ receptor and other receptors (Chen et al., 2011; Cook et al., 2008; Laínez et al., 2010; Leil et al., 2004). Therefore, we next investigated the involvement of GABARAP in insulin secretion by silencing *Gabarap* expression using *Gabarap*-siRNA (1) and *Gabarap*-siRNA (2). Successful *Gabarap* knockdown was confirmed by western blotting (supplementary material Fig. S1C) and immunocytochemistry (supplementary material Fig. S4). As shown in Fig. 4A, in response to high glucose stimulation, higher levels of insulin release were observed in cells at 4–7 min and 7–10 min than at basal level (−2–0 min). Compared to the control cells, insulin secretion from the cells transfected with *Gabarap*-siRNA (1), was significantly decreased at both 4–7 min and 7–10 min (36% and 30% reduction, respectively), and a similar result was obtained in MIN6 cells transfected with *Gabarap*-siRNA (2) (supplementary material Fig. S1D). *Prip* knockdown reversed the decreased insulin secretion from *Gabarap*-siRNA (1)-transfected cells to the level observed in the control cells only during the 7–10 min time point (Fig. 4A). Furthermore, the accumulated distance traveled by the secretory vesicles was also reduced by *Gabarap* knockdown under high glucose conditions, which was partially potentiated by additional *Prip* knockdown (Fig. 4B, left panel). These results could be interpreted as follows: following additional *PRIP*-knockdown, the endogenous GABARAP remaining in cells after *Gabarap* silencing became free and promoted insulin secretion and vesicle mobility. In turn, *Prip1* overexpression in MIN6 cells decreased the mobility of insulin vesicles, and this effect was further inhibited by transfection with *Gabarap*-siRNA (Fig. 4B, right panel). These data suggest that a complex of PRIP and GABARAP regulates KIF5B-mediated insulin vesicle transport; GABARAP appears to promote vesicle trafficking, whereas PRIP blocks this effect.

To investigate the effect of *Gabarap* silencing, we examined the co-localization of insulin vesicles with KIF5B and microtubules under high glucose conditions. *Gabarap* knockdown had little effect on the localization of KIF5 with β-tubulin (Fig. 4C,F). However, insulin vesicles co-localized with KIF5 or β-tubulin to a lesser extent following *Gabarap* knockdown (for KIF5 and β-tubulin, see Fig. 4D,G and Fig. 4E,H, respectively). Consistently, silencing of *Kif5b* had little effect on the localization of GABARAP and insulin vesicles (supplementary material Fig. S3C). These data suggest that GABARAP facilitates localization of insulin vesicles on microtubules, probably through its binding to insulin vesicles and microtubules; thus, GABARAP presents insulin vesicles to KIF5. Since GABARAP associates with the microtubules through its microtubule-binding domain (amino acid residues 1–22) (Wang and Olsen, 2000), we next examined co-localization using a plasmid expressing a GABARAP protein (GABARAP2–35) that inhibits the binding of GABARAP with tubulin. As shown in Fig. 4I,J, the co-localization of insulin vesicles with KIF5 was significantly inhibited in cells expressing GABARAP2–35 peptide, suggesting the importance of the interaction between GABARAP and microtubules for the co-localization of insulin vesicles with KIF5.

**Fig. 5.**
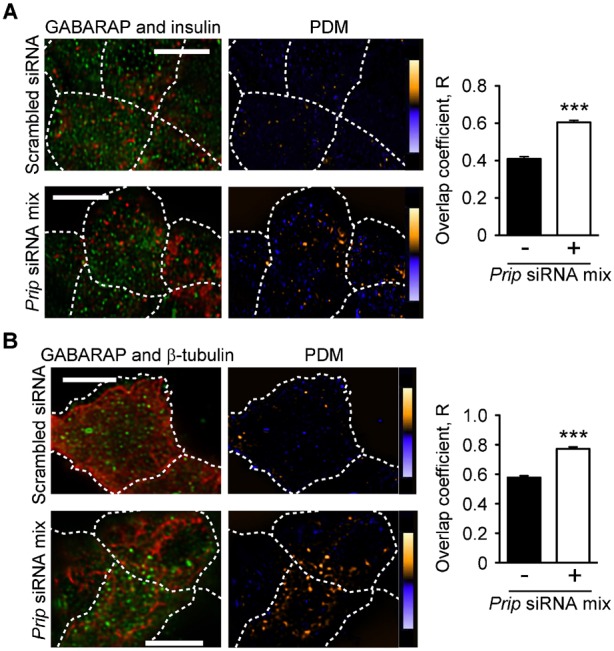
*Prip* silencing enhances the co-localization of GABARAP with insulin vesicles and β-tubulin. Co-localization of GABARAP (green) with insulin (red) (A) or β-tubulin (red) (B) in MIN6 cells transfected with scrambled siRNA (upper panels) or *Prip* siRNA mix [*Prip1*-siRNAs (1, 2, 3) and *Prip2*-siRNAs (1, 2, 3), lower panels] after stimulation with 30 mM glucose for 10 min. Magnified images of panels A and B are shown in supplementary material Fig. S9B,C. A set of representative images from 3 independent experiments is shown. The overlap coefficient was calculated. Values are presented as mean ± s.d. (from left in each graph of panels A and B; *n* = 144 and 112; 84 and 160, respectively); ***p<0.001. Scale bars: 5µm.

We next examined the effect of *Prip* silencing on GABARAP functions. In *Prip*-knockdown MIN6 cells under high glucose conditions, GABARAP was highly co-localized with insulin vesicles and β-tubulin (see PDM panels in Fig. 5A and Fig. 5B, respectively) compared to the control cells. Consistently, fractionation analysis using an OptiPrep™ discontinuous gradient revealed that the GABARAP signal in the secretory vesicle fractions was faint in control cells under low glucose conditions, but was higher in *Prip*-knockdown cells under both low and high glucose conditions (supplementary material Fig. S5A,B). These results suggest that PRIP negatively regulates the association of GABARAP with insulin vesicles and microtubules in MIN6 cells.

### A GABARAP peptide that interferes with its binding to PRIP mediates insulin vesicle localization with microtubules and KIF5, which facilitates insulin vesicle transport

**Fig. 6.**
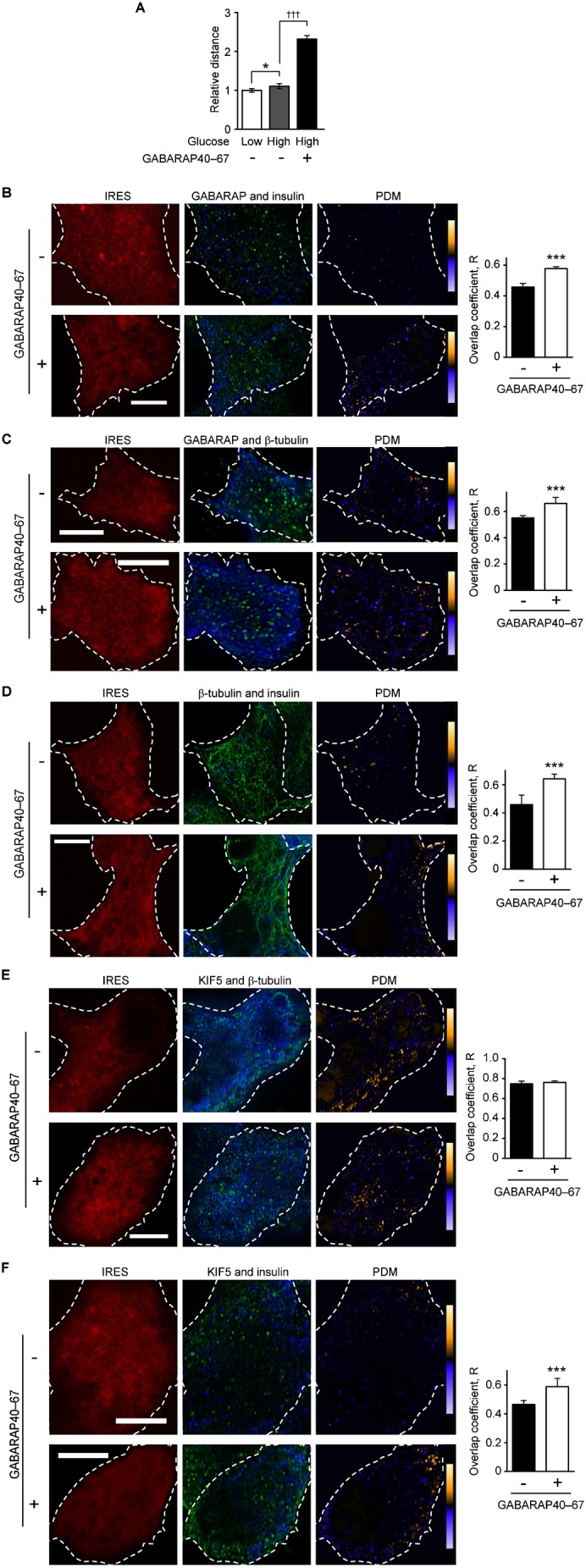
Overexpression of GABARAP40–67 promotes the co-localization of GABARAP with insulin vesicles, microtubules, and KIF5, and facilitates insulin vesicle movements. (A) Quantitative analysis of insulin vesicle movement in MIN6 cells transfected with or without pIRES2-DsRed/GABARAP40–67. The accumulated distance of a randomly selected track of insulin vesicles was calculated. Values are presented as mean ± s.e. (from the left, *n* = 214, 233, and 326, respectively). (B–F) Co-localization analyses of GABARAP (green) and insulin (blue) (B), GABARAP (green) and β-tubulin (blue) (C), β-tubulin (green) and insulin (blue) (D), KIF5 (green) and β-tubulin (blue) (E), and KIF5 (green) and insulin (blue) (F) in MIN6 cells transfected with pIRES2-DsRed/empty (upper panels) or pIRES2-DsRed/GABARAP40–67 (lower panels) in response to stimulation with 30 mM glucose for 10 min. Endogenous GABARAP was stained with an anti-GABARAP antibody, which recognized amino acid residues 1–39 of GABARAP. Magnified images of B–F are shown in supplementary material Fig. S10A–E. A set of typical images from 3 independent experiments is shown. The dotted line shows a cell edge. The overlap coefficient in each co-localization experiment was calculated. Values are presented as mean ± s.d. [from the left in each graph, *n* = 64 and 76 (B); 40 and 40 (C); 40 and 40 (D); 52 and 60 (E); and 40 and 60 (F)]. *p<0.05, ***p<0.001, ^†††^*p*<0.001. Scale bars: 5 µm.

The PRIP–GABARAP complex may regulate KIF5B-mediated insulin vesicle transport. PRIP binds to GABARAP at amino acid residues 40–68, and a peptide that includes these residues inhibits binding (Kanematsu et al., 2002; Kanematsu et al., 2005; Kanematsu et al., 2006; Mizokami et al., 2007). Therefore, we used this peptide to examine the importance of this association for insulin secretion. PRIP1 co-precipitated with HaloTag®-fused GABARAP40–67, but not with control IgG (supplementary material Fig. S5C), indicating that the GABARAP40–67 peptide associates with PRIP. Introduction of a pIRES2-DsRed vector containing GABARAP40–67 into cells triggered dissociation of PRIP from GABARAP and increased insulin vesicle mobility in response to high glucose stimulation (Fig. 6A).

To determine if dissociation of GABARAP from PRIP regulates the co-localization of insulin vesicles with microtubules, we transfected pIRES2-DsRed/GABARAP40–67 into MIN6 cells and performed immunocytochemical analyses after high glucose stimulation. Compared to cells transfected with the empty vector, GABARAP was highly co-localized with insulin vesicles and β-tubulin (see the PDM panels and graph in Fig. 6B,C). Consequently, co-localization of insulin vesicles with β-tubulin was promoted in GABARAP40–67-expressing MIN6 cells (see PDM panel and graph in Fig. 6D), suggesting that GABARAP freed from PRIP facilitates bridging between insulin vesicles and microtubules. Although transfection of GABARAP40–67 had no effect on the localization of KIF5 with β-tubulin (see PDM panel and graph in Fig. 6E), it significantly increased the co-localization of KIF5 and insulin vesicles (see PDM panel and graph in Fig. 6F). To further confirm the effect of GABARAP–PRIP dissociation, we examined the levels of KIF5 and GABARAP in the vesicle fraction (fraction 11 in Fig. 3A) using OptiPrep™ step-gradient analysis. Transfection of GABARAP40–67 into MIN6 cells promoted the accumulation of KIF5 (supplementary material Fig. S5D,E) and GABARAP (supplementary material Fig. S5D,F) in the vesicle fractions.

**Fig. 7.**
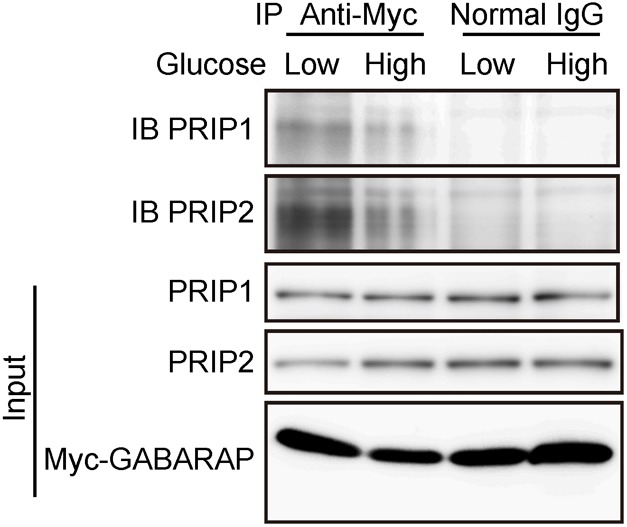
Dissociation of GABARAP from PRIP in response to glucose stimulation. MIN6 cells transfected with myc-tagged GABARAP were stimulated with low (5 mM) or high glucose (30 mM), followed by immunoprecipitation with an anti-myc antibody or control IgG. Immunoprecipitates were analyzed by SDS-PAGE and western blotting using the indicated primary antibodies. Similar results were obtained from 3 independent experiments.

To elucidate whether PRIP and GABARAP interaction is regulated by extracellular glucose concentration, we performed an OptiPrep™ discontinuous gradient fractionation assay (supplementary material Fig. S5G) following incubation of cells in high and low glucose. More GABARAP was accumulated in the insulin vesicle fraction in high glucose than in low glucose. Furthermore, to determine if the amount of PRIP bound to GABARAP is altered in response to high glucose stimulation, we performed an immunoprecipitation assay using MIN6 cells transfected with a myc-tagged GABARAP plasmid (Fig. 7). Under low glucose conditions, PRIP1 and PRIP2 were immunoprecipitated by an anti-myc antibody, whereas the amounts of PRIP1 and PRIP2 precipitated were significantly decreased following high glucose stimulation, suggesting that extracellular glucose regulates the association of GABARAP with PRIP.

### PRIP deficiency increases the glucose-induced second phase of insulin secretion in pancreatic islets

**Fig. 8.**
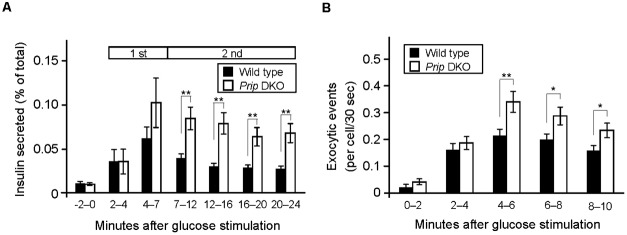
PRIP deficiency increases glucose-induced insulin secretion and exocytic events in pancreatic islets. (A) Time course of glucose-stimulated insulin secretion from *Prip*-DKO and wild-type mouse pancreatic islets. Isolated pancreatic islets were stimulated with 20 mM glucose, and the amount of insulin released was measured. Insulin secretion was normalized to intracellular insulin content, and is presented as a percentage of the total intracellular content. Insulin secretion per min was compared during the indicated periods. The first and second phases were defined as 2–7 min, and over 7 min, respectively. Values are presented as mean ± s.e. (*n* = 5 for each genotype). (B) Time course of exocytic events observed by two-photon microscopy during glucose stimulation (20 mM) in pancreatic islets from *Prip*-DKO and wild-type mice. More than 400 exocytic events from >100 cells of each genotype in 5 independent experiments were analyzed. The number of exocytic events is expressed as events per cell per 30-sec interval and is shown as the mean number of events during the indicated time periods. Values are presented as mean ± s.e. (wild type, *n* = 126; *Prip*-DKO, *n* = 116). *p<0.05, **p<0.01.

To confirm the physiological regulation of insulin exocytosis by PRIP, we performed an insulin perfusion assay using islets of Langerhans prepared from *Prip*-DKO pancreas. Both PRIP1 and PRIP2 were expressed in isolated wild-type pancreatic islets (supplementary material Fig. S6A). In response to stimulation with 20 mM glucose, insulin secretion was rapidly initiated (with a delay of 2 min), and was followed by long-lasting attenuating secretion. Biphasic insulin secretion is characterized as having a transient first phase that lasts for 2–7 min and a sustained second phase, which occurs over the next 7 min (Rorsman and Renström, 2003). Similar levels of insulin were released from islets isolated from wild-type and *Prip*-DKO mice in the first phase (2–7 min), whereas secretion in the second phase was significantly increased (approximately 1.9–2.6-fold) in *Prip*-DKO mice compared to wild-type mice (Fig. 8A). To analyze individual exocytic events, we conducted live imaging by using two-photon microscopy. Pancreatic islets from *Prip*-DKO and wild-type mice were stimulated by exposure to 20 mM glucose, and the abrupt appearance of small fluorescent spots (Ω-like structures) in the intracellular area were analyzed as previously described (Hatakeyama et al., 2006; Takahashi et al., 2002). The event number and occurrence time were counted, and the rate of insulin exocytosis was calculated. The number of exocytic events in the initial phase (0–4 min) did not differ between *Prip*-DKO and wild-type mice. However, insulin secretion events in *Prip*-DKO cells were significantly higher late in the first phase (4–6 min) and during the sustained second phase (6–8 min and 8–10 min) than in wild-type mice (Fig. 8B). We also quantified the distribution of maximum intensity (supplementary material Fig. S6B) and the lifetime of a secretory granule (expressed in terms of time before [T1] and after [T0] reaching peak intensity; supplementary material Fig. S6C,D), indicating that *Prip* deficiency may not affect the vesicle size and fusion kinetics of insulin granules. These results suggest that *Prip* knockout increases the insulin exocytic events during the second phase of release, but not the vesicle size and fusion kinetics of insulin granules in pancreatic islets.

In pancreatic β-cells, insulin secretion following granule fusion is triggered by extracellular Ca^2+^ entry via a voltage-dependent Ca^2+^ channel (Prentki and Matschinsky, 1987). We examined the role of PRIP in glucose-induced Ca^2+^ influx by simultaneous imaging of Ca^2+^ signals (supplementary material Fig. S7A). However, there were no significant differences in the mean values of Ca^2+^ influx onset time (approximately 65 sec; supplementary material Fig. S7B), maximum Ca^2+^ concentration (supplementary material Fig. S7C), and slope (Ca^2+^ increase rate; supplementary material Fig. S7D) between the genotypes. These data suggest that PRIP affects second-phase insulin release without influencing the process of glucose stimulation-induced Ca^2+^ influx (i.e. glucose metabolism followed by voltage-gated Ca^2+^ channel opening).

## DISCUSSION

Insulin-containing vesicle transport is believed to involve long-range movement of the cargo by kinesin on microtubules (Balczon et al., 1992). In this study, we demonstrated that PRIP is a novel modulator of vesicle–kinesin complex formation. This complex consists of the motor protein KIF5B, the vesicle trafficking modulator GABARAP, β-tubulin (microtubules), and insulin-containing secretory vesicles. PRIP deficiency results in the localization of insulin granules with KIF5B and acceleration of vesicle trafficking. We also showed that GABARAP was involved in KIF5B-mediated vesicle trafficking as a molecule tethering the secretory vesicles to KIF5B and microtubules. These findings illuminate a novel mechanism in the regulation of insulin vesicle trafficking.

We demonstrated that PRIP and GABARAP act as a negative and a positive modulator, respectively, in the insulin secretory pathway by gene silencing with specific siRNAs in MIN6 cells. Since GABARAP is a PRIP binding partner and a modulator of receptor trafficking (Chen et al., 2011; Cook et al., 2008; Kanematsu et al., 2002; Laínez et al., 2010; Leil et al., 2004; Mizokami et al., 2007), understanding the molecular relationship between PRIP and GABARAP provides new insight into how insulin vesicle transport and secretion are regulated.

GABARAP has the ability to interact with microtubules *in vivo* (Wang et al., 1999; Wang and Olsen, 2000) and promote tubulin polymerization *in vitro* (Coyle et al., 2002; Wang and Olsen, 2000). There have been several reports that disruption of microtubule polymerization inhibits the sustained phase of insulin secretion (Farshori and Goode, 1994; Howell et al., 1982). Cytoskeletal motors such as kinesin, a two-headed processive motor, are capable of taking sequential steps along polymerized microtubules by direct binding. Many motors have been found to use an accessory protein to provide “secondary binding sites” that aid in motor action processivity (Kincaid and King, 2006). GABARAP is not a motor protein; however, GABARAP could function as a molecule that helps tether the kinesin–vesicle complex on the microtubules (Cook et al., 2008). We showed that, in addition to *Prip*-knockdown experiment, dissociation of PRIP from GABARAP by GABARAP40–67 facilitated the localization of GABARAP to the microtubules, insulin vesicles, and KIF5 (Fig. 5B, Fig. 6C,F), which enhanced insulin vesicle transport (Fig. 1E, Fig. 6A) and insulin secretion (Fig. 1B). Interestingly, glucose stimulation disrupted the binding of GABARAP with PRIP (Fig. 7). *Gabarap*-knockdown inhibited the co-localization of insulin vesicles with KIF5 (Fig. 4D,G) and attenuated vesicle transport and subsequent insulin secretion (Fig. 4A,B). Moreover, the dissociation of GABARAP from microtubules by an inhibitory peptide (myc-GABARAP2–35) significantly inhibited the co-localization of KIF5B and insulin vesicles (Fig. 4I,J). These data suggest that, in response to high extracellular glucose, GABARAP is freed from PRIP, is localized to microtubules, and tethers insulin vesicles to kinesin, allowing kinesin to move insulin vesicles to the cell periphery, which upregulates insulin secretion.

Glucose stimulation induces the disruption of actin filaments in primary β-cells and MIN6 cells (Nevins and Thurmond, 2003; Tomas et al., 2006). Disruption of a dense web, consisting of actin filaments, leads to dramatic increases in insulin secretion in the first phase (Howell and Tyhurst, 1979; Orci et al., 1972; Van Obberghen et al., 1973; Wang et al., 1990), suggesting that actin filaments impede the access of insulin granules to the cell periphery. Glucose stimulation has been shown to transiently disrupt the interaction of filamentous actin (F-actin) with t-SNARE proteins at the plasma membrane in primary β-cells and MIN6 cells (Thurmond et al., 2003). This disruption promotes insulin secretion, which is mediated by increased granule accumulation at the plasma membrane and increased t-SNARE accessibility (Jewell et al., 2008). In our study, GABARAP deficiency had an effect on insulin release in both the first phase (4–7 min) and the second phase (>7 min) after glucose stimulation. It was previously reported that GABARAP binds to microfilaments (actin) as well as microtubules (β-tubulin); the actin binding is not direct, and it may be mediated by yet unknown proteins (Wang and Olsen, 2000). Insulin vesicles move along microtubules to the cell surface, and are then transported to actin filaments by switching to myosin-driven transport (Varadi et al., 2003). Therefore, these data suggest that GABARAP may affect insulin release by regulating both microtubule- and actin filament-mediated secretory pathways.

Gao et al. recently reported that PRIP regulates the process of exocytosis that is modulated by the phospho-state of SNAP-25 in PC12 cells (Gao et al., 2012). Because PC12 cells do not express intrinsic PRIP1 and PRIP2, the authors exogenously transfected *Prip1* and measured adrenaline secretion. PRIP overexpression inhibited adrenaline release 20–30 min after forskolin stimulation, but did not inhibit release occurring less than 10 min after stimulation. However, little is known about glucose-induced insulin secretion via PRIP-mediated SNAP-25 phosphoregulation in pancreatic β-cells, and the involvement of PRIP in SNARE complex-regulated exocytosis remains to be confirmed.

Taken together, this study extends our understanding of the physiological regulation of PRIP and suggests a novel function for GABARAP in insulin exocytosis. PRIP negatively regulates the insulin secretory pathway by inhibiting the formation of a complex among GABARAP, insulin vesicles, and microtubules. Furthermore, GABARAP facilitates the anchoring of KIF5 associated with insulin granules on microtubules leading to insulin vesicle trafficking. In conclusion, the PRIP–GABARAP complex-regulated vesicle trafficking system constitutes novel secretory machinery for insulin exocytosis. This finding may provide new therapeutic approaches for diabetes, such as β-cell implantation. It may be possible to produce a pancreatic β-cell that efficiently secretes insulin by silencing *PRIP* or by transfecting an interference peptide targeted at the interaction between PRIP and GABARAP.

## Supplementary Material

Supplementary Material
