# Supplementary material for: Phospholipase C-related catalytically inactive protein (PRIP) controls KIF5B-mediated insulin secretion
Source: Biol Open. 2014 May 8;3(6):463–74. doi: 10.1242/bio.20147591 (PMC4058080; doi:10.1242/bio.20147591)
Supplement: Supplementary Material [file supp_3_6_463__index.html]

Phospholipase C-related catalytically inactive protein (PRIP) controls KIF5B-mediated insulin secretion — Supplementary Material 

# Phospholipase C-related catalytically inactive protein (PRIP) controls KIF5B-mediated insulin secretion

## bio.20147591 Supplementary Material

**Files in this Data Supplement:**

- Supplementary Material - Satoshi Asano et al. doi: 10.1242/bio.20147591
