# Supplementary material for: Phospholipase C-related catalytically inactive protein (PRIP) controls KIF5B-mediated insulin secretion
Source: Biol Open. 2014 May 8;3(6):463–74. doi: 10.1242/bio.20147591 (PMC4058080; doi:10.1242/bio.20147591)
Supplement: Supplementary Material [file supp_bio.20147591_bio.20147591-s1.pdf]

Biology Open (2014) 000, 1–12 doi:10.1242/bio.20147591

Supplementary Material

Satoshi Asano et al. doi: 10.1242/bio.20147591

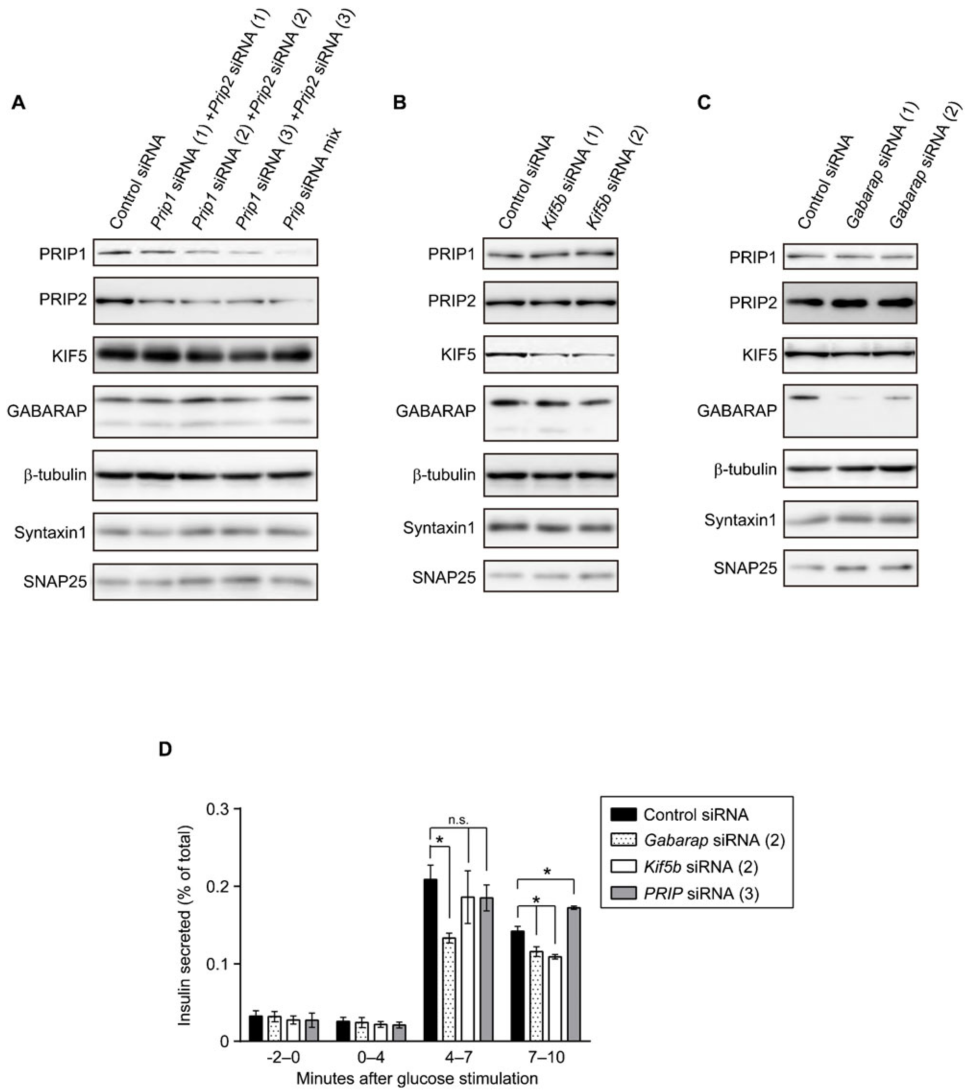

Fig. S1. Silencing of *Prip*, *Kif5b*, and *Gabarap* in MIN6 cells. (A–C) Western blot analyses. MIN6 cells were transfected with the indicated siRNA(s): a control siRNA, a pair of *Prip1*- and *Prip2*-siRNAs, or a *Prip*-siRNA mix [a mixture of *Prip1*-siRNAs (1, 2, 3) and *Prip2*-siRNAs (1, 2, 3)] (A), *Kif5b*-siRNA (1) and *Kif5b*-siRNA (2) (B), and *Gabarap*-siRNA (1) and *Gabarap*-siRNA (2) (C), and were cultured for 2 days. Whole cell lysates were analyzed by western blotting. Equivalent amounts of protein were loaded into each well (see β-tubulin staining). The expression level of each target protein was lower in their respective siRNA-transfected cells than that in the control siRNA-transfected cells, whereas untargeted protein expression levels were unchanged. In addition, since PRIP regulates soluble N-ethylmaleimide-sensitive factor attachment protein receptor (SNARE) functions, we examined syntaxin1 and SNAP25 expression. The expression levels of syntaxin1 and SNAP25 in all knockdown cells examined were unchanged. We obtained similar results from 3 independent experiments, and a set of typical images is shown. (D) Insulin secretion assay using MIN6 cells. Cells were transfected with *Gabarap*-siRNA (2), *Kif5b*-siRNA (2), or *Prip1*-siRNA (3) and *Prip2*-siRNA (3), stimulated with 30 mM glucose, and then released insulin was measured every 1 min. Insulin secretion was normalized to intracellular insulin content, and is presented as a percentage of the total intracellular content. Values are presented as mean ± s.e. ($n=3$); *$p<0.05$; n.s., not statistically significant.

Biology Open

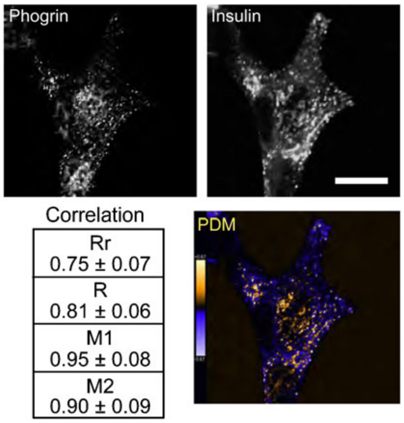

Fig. S2. Co-localization of phogrin and insulin-containing vesicles in MIN6 cells. Cells transfected with GFP-phogrin were fixed with 3.7% paraformaldehyde, subjected to immunocytochemistry with an anti-insulin antibody, and processed for confocal microscopy to detect phogrin (upper left panel) and insulin (upper right panel). The yellow and blue pseudo-colors in the PDM image show areas of high and low co-localization, respectively. Co-localization was analyzed by ImageJ intensity correlation analysis. Rr, Pearson's correlation coefficient; R, Mander's overlap coefficient; M1, Mander's co-localization coefficient for insulin; M2, Mander's co-localization coefficient for phogrin. We performed these experiments 3 times and obtained similar results. Scale bar: 5 µm.

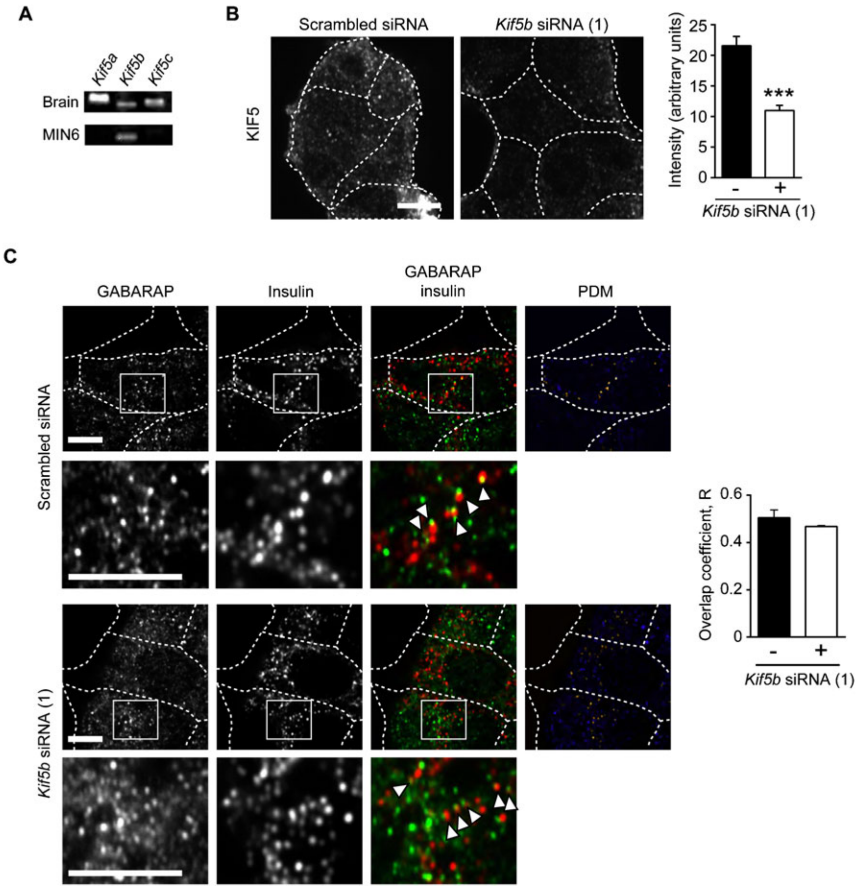

Fig. S3. Silencing of *Kif5b* in MIN6 cells. (A) KIF5 subtype expression in MIN6 cells. The mRNA expression of *Kif5a*, *Kif5b*, and *Kif5c* in the mouse brain and MIN6 cells was analyzed by reverse transcription-polymerase chain reaction. (B) KIF5 expression in MIN6 cells. Cells transfected with or without *Kif5b*-siRNA (1) were fixed with 3.7% paraformaldehyde, subjected to immunocytochemistry with an anti-KIF5 antibody, and processed for confocal microscopy. The dotted line shows a cell edge. The graph shows the mean fluorescent intensity (arbitrary units) of KIF5 per cell. Values are presented as mean ± s.d. (n=40); ***p<0.001. (C) Co-localization of GABARAP (green) and insulin (red) in MIN6 cells transfected with either scrambled siRNA or *Kif5b*-siRNA (1). After a 10-min exposure to 30 mM glucose, cells were fixed with 3.7% paraformaldehyde for confocal microscopic observation. The yellow and blue pseudo-colors in the PDM images show areas of high and low co-localization, respectively. A set of typical images from 3 independent experiments is shown. The dotted line shows a cell edge. Each lower panel shows a magnified view of the framed area. The graph shows the overlap coefficient. Values are presented as mean ± s.d.; the difference is not statistically significant (from the left, n=114 and 195, respectively). Scale bars: 5 µm.

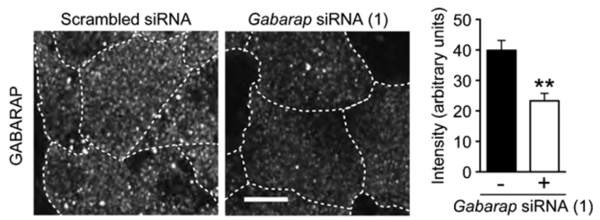

Fig. S4. Silencing of GABARAP in MIN6 cells. Cells transfected with or without *Gabarap*-siRNA were subjected to immunocytochemistry with an anti-GABARAP antibody. The dotted line shows a cell edge. The graph shows the mean fluorescent intensity (arbitrary units) of GABARAP per cell. Values are presented as mean ± s.d. (n=20); **p<0.01. Scale bar: 5 μm.

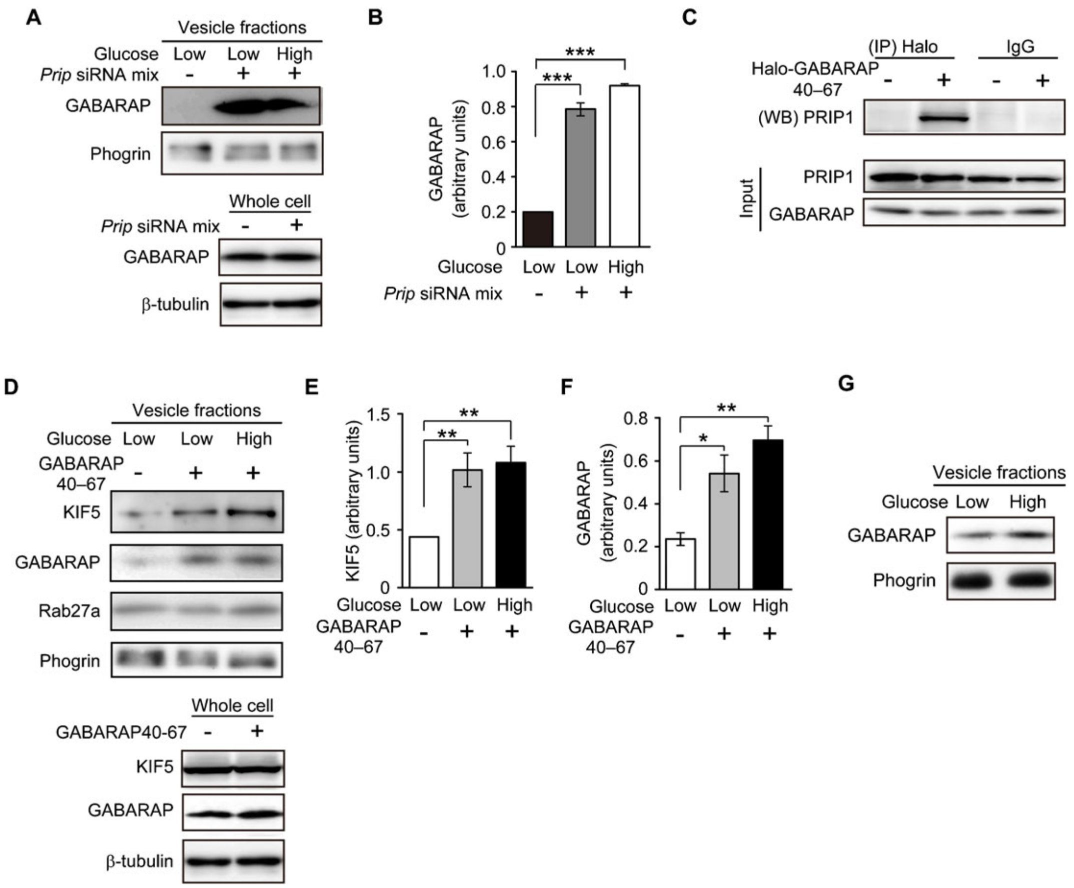

Fig. S5. Analyses of the involvement of GABARAP in insulin secretion. (A,B) MIN6 cells were transfected with scrambled siRNA (−) or *Prip*-siRNA mix [*Prip1*-siRNAs (1, 2, 3) and *Prip2*-siRNAs (1, 2, 3)]. Two days after transfection, cultured cells were stimulated with or without 30 mM glucose for 10 min, and extracted with homogenization buffer. Equivalent protein amounts in the cell homogenates were fractionated in OptiPrep™ discontinuous gradients (3%, 7.5%, 18%, and 35%). The vesicle fraction [fraction 11 (Fig. 3)] was collected, and GABARAP content was analyzed by western blotting (A). The total amount of GABARAP in the whole cell homogenates from *Prip*-knockdown and control cells did not differ (lowest blot in panel A). Three independent experiments were performed, and a set of typical images is shown. The mean values of the GABARAP bands are shown in the graph (B). Values are presented as mean ± s.d. (n=3); ***p<0.001. (C) A Halo-GABARAP40–67 peptide binds PRIP1. MIN6 cells transfected with a Halo-GABARAP40–67-plasmid or an empty vector were solubilized, and the resulting extract was immunoprecipitated with an anti-Halo antibody or control IgG. Then, western blotting was performed using an anti-PRIP1 antibody. Similar results were obtained in 3 additional experiments, and a set of typical images is shown. (D–F) Accumulation of GABARAP and KIF5 in insulin vesicle fractions from MIN6 cells transfected with GABARAP40–67. Cells were transfected with pIRES2-DsRed/GABARAP40–67, and then stimulated with 30 mM glucose for 10 min. Cell lysates were fractionated using OptiPrep™ discontinuous gradients (3%, 7.5%, 18%, and 35%). The secretory vesicle-rich fractions were analyzed by western blotting. Similar results were obtained from 3 independent experiments, and a set of typical images is shown (D). The total amounts of KIF5 and GABARAP in GABARAP40–67-transfected and control cells are similar (lowest blot in panel D). The density of each band was calculated, and the values of KIF5 (E) and GABARAP (F) are presented as arbitrary units based on phogrin density. Values are presented as mean ± s.d. (n=3); *p<0.05, **p<0.01. (G) Accumulation of GABARAP in insulin vesicle fractions from MIN6 cells after high glucose stimulation. The insulin vesicle fraction was prepared by fractionation using an OptiPrep™ discontinuous gradient, and western blotting was performed. Similar results were obtained from 3 independent experiments.

Biology Open

Biology Open (2014) 000, 1–12 doi:10.1242/bio.20147591

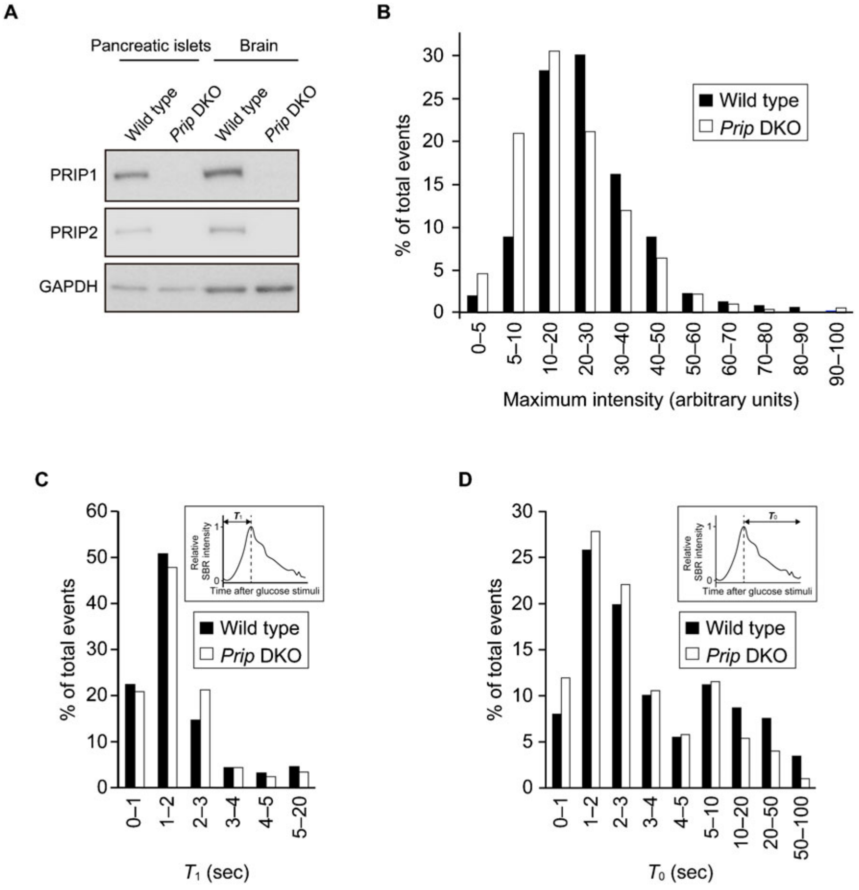

Fig. S6. Characterization of the fusion events during insulin secretion by two-photon imaging analysis using pancreatic islets. (A) PRIP expression in pancreatic islets. Pancreatic islets were enzymatically isolated from wild-type and *Prip*-DKO mouse pancreas. Homogenates of the isolated islets and a piece of cerebral cortex were analyzed by western blotting using anti-PRIP1 and anti-PRIP2 antibodies.
(B–D) Characteristics of exocytic events in the islets isolated from wild-type and *Prip*-DKO mice were examined by two-photon microscopic observation. We analyzed 438 and 501 events in wild-type and *Prip*-DKO cells, respectively, in 5 independent experiments. The distribution of the maximum fluorescence intensity in each Ω-like profile (B). The time to reach the maximum intensity after glucose stimulation and the time to return to basal levels were calculated and shown in panels C and D, respectively.

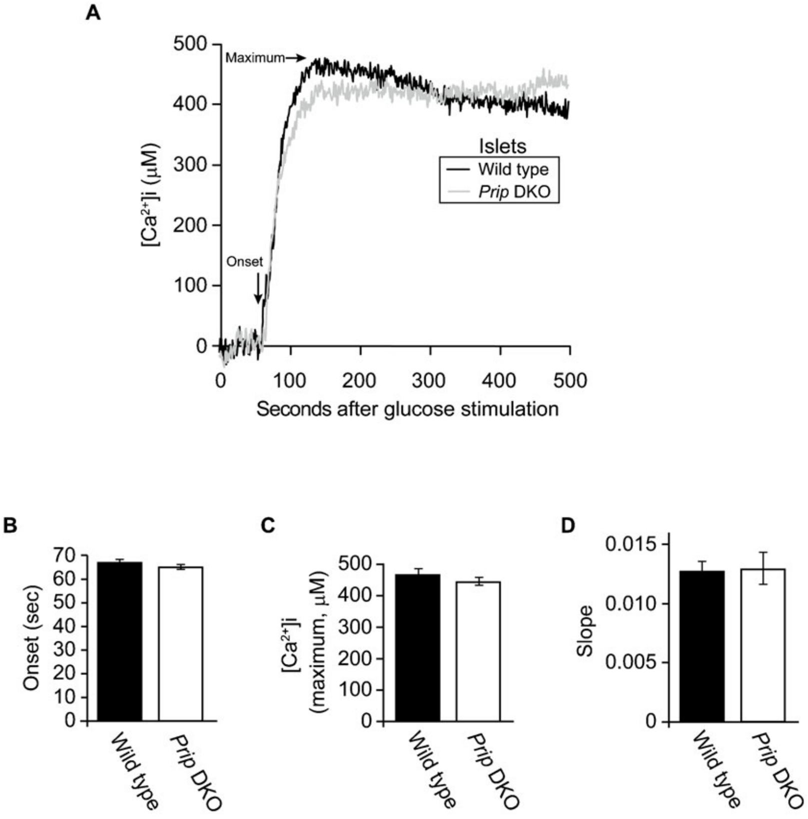

Fig. S7. Comparison of the glucose-induced Ca^{2+} responses between wild-type and *Prip*-DKO cells. Two-photon calcium imaging was simultaneously performed with pancreatic islets isolated from wild-type and *Prip*-DKO mice, when the insulin exocytic events were visualized by high glucose stimulation (20 mM). Islets were loaded with the Ca^{2+} indicator fura-2-AM (10 μM) for 40 min at 37˚C. Relative Ca^{2+} responses from more than 49 cells were analyzed in 3 independent experiments, and a set of typical Ca^{2+} responses is shown (A). Onset time (B), the relative maximum Ca^{2+} response (C), and the slope (from initiation until the point at which the maximal Ca^{2+} concentration was obtained) (D) were examined. Values are presented as mean ± s.d. (wild type, n=60; *PRIP*-DKO, n=49).

Biology Open (2014) 000, 1–12 doi:10.1242/bio.20147591

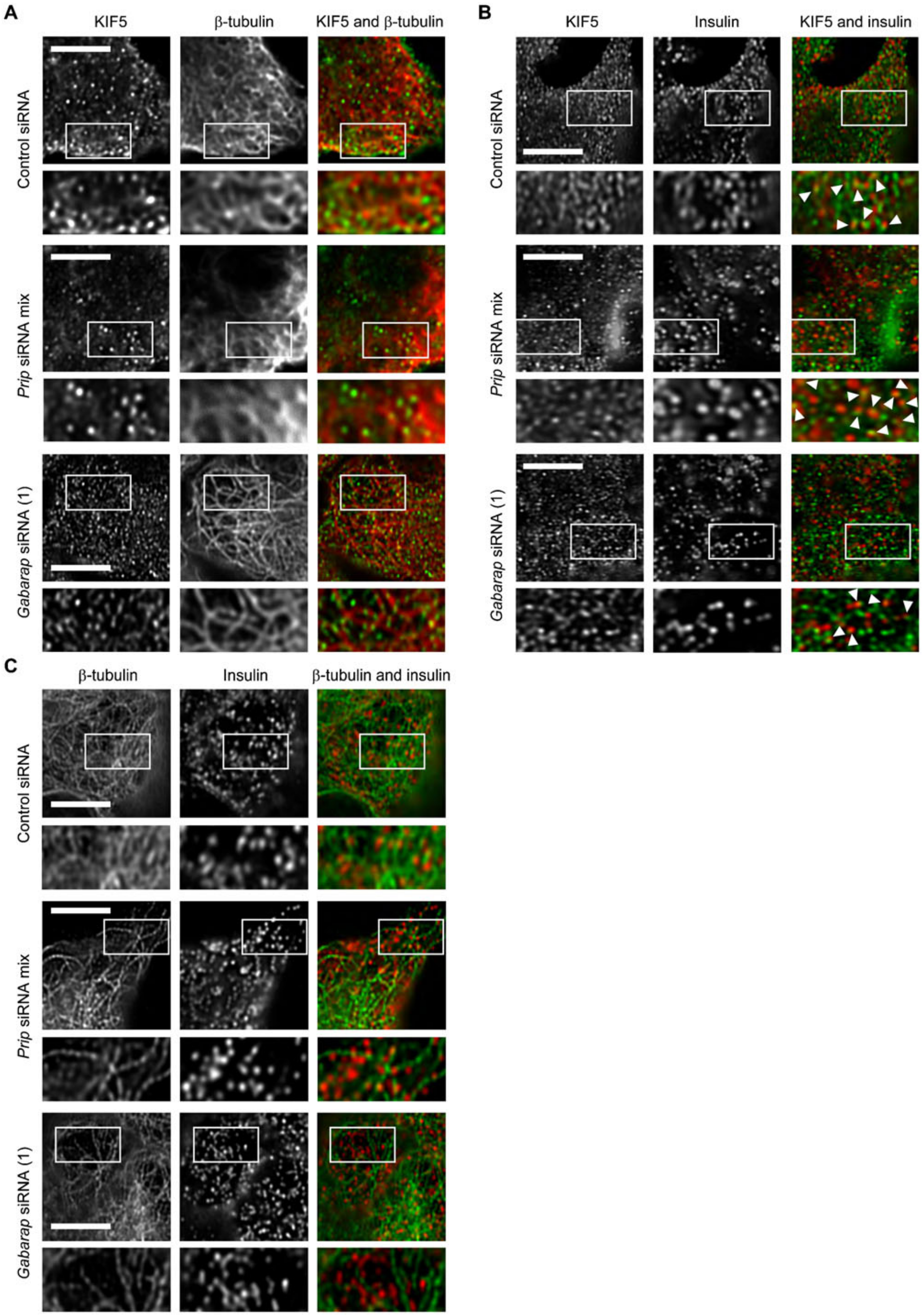

Fig. S8. KIF5, β-tubulin, and insulin localization in MIN6 cells transfected with *Prip*-siRNA or *Gabarap*-siRNA. Co-localization of KIF5 (green) and β-tubulin (red) (A), KIF5 (green) and insulin (red) (B), and β-tubulin (green) and insulin (red) (C) in MIN6 cells transfected with control siRNA (upper 2 panels) and *Prip*-siRNA mix [*Prip1*-siRNAs (1, 2, 3) and *Prip2*-siRNAs (1, 2, 3)] (middle 2 panels) or *Gabarap*-siRNA (1) (lower 2 panels). After a 10-min exposure to 30 mM glucose, cells were fixed with 3.7% paraformaldehyde for confocal microscopic observation. The representative cells shown here are different from those shown in Fig. 2C–E and Fig. 4C–E. The framed area is magnified below in each set. Arrowheads indicate the co-localization of KIF5 and insulin (B). A set of typical images from more than 45 cells in 3 independent experiments is shown. Scale bars: 5 µm.

Biology Open (2014) 000, 1–12 doi:10.1242/bio.20147591

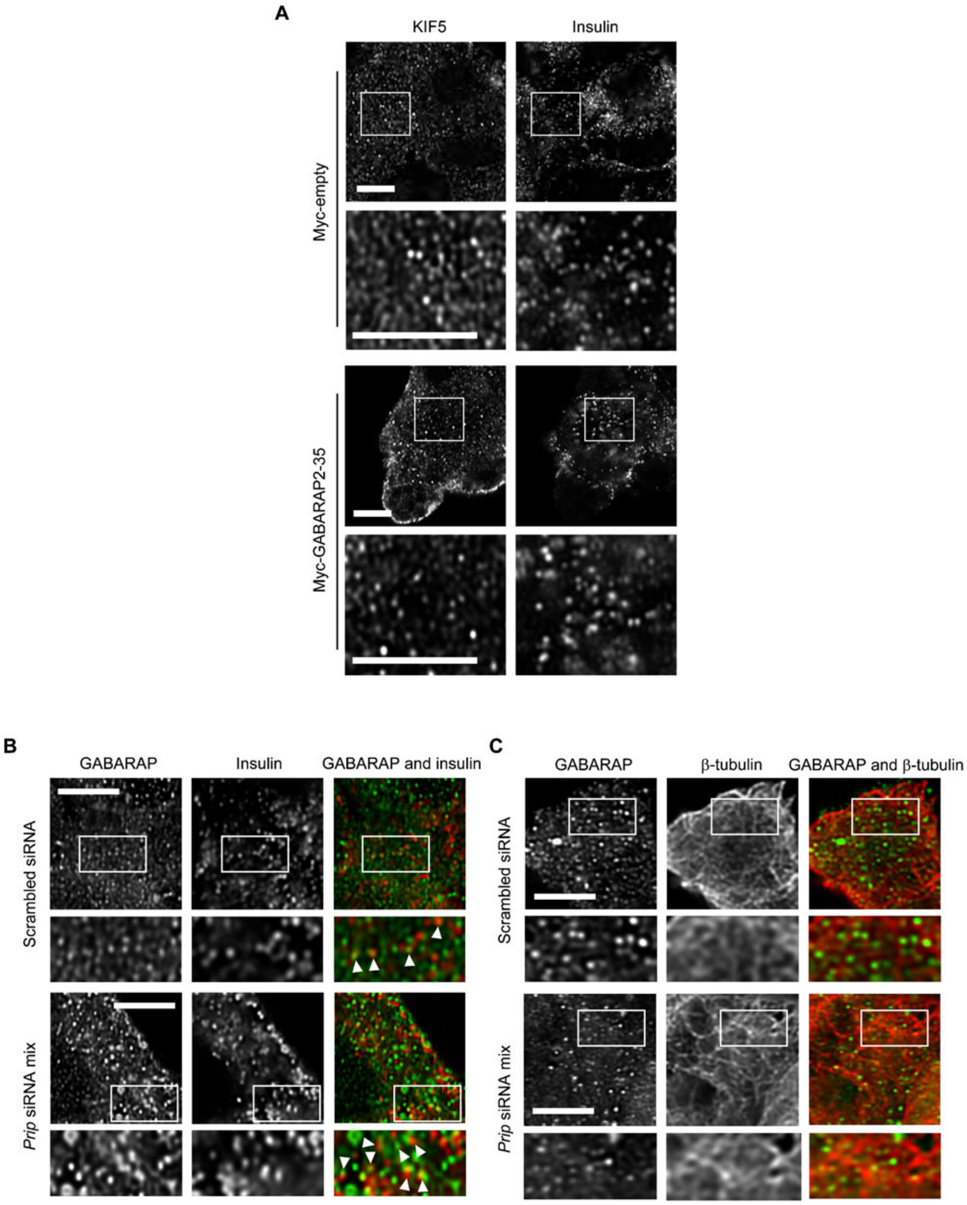

Fig. S9. Immunocytochemical analysis of MIN6 cells transfected with myc-GABARAP2–35 or *Prip* siRNA mix. Co-localization of KIF5 and insulin in MIN6 cells (A) transfected with a myc-empty vector (upper panels) or myc-GABARAP2–35 (lower panels), GABARAP (green) and insulin (red) (B), or β-tubulin (red) (C) in MIN6 cells transfected with scrambled siRNA (B,C, upper panels) or *Prip*-siRNA mix [*Prip1*-siRNAs (1, 2, 3) and *Prip2*-siRNAs (1, 2, 3) (B,C, lower panels)]. After a 10-min exposure to 30 mM glucose, cells were fixed with 3.7% paraformaldehyde for confocal microscopy. The representative cells shown here are different from those shown in Fig. 5A,B (B,C). The framed area is magnified below. A set of typical images from more than 81 cells in 3 independent experiments is shown. Arrowheads indicate the co-localization of GABARAP with insulin (B). Scale bars: 5 µm.

Biology Open (2014) 000, 1–12 doi:10.1242/bio.20147591

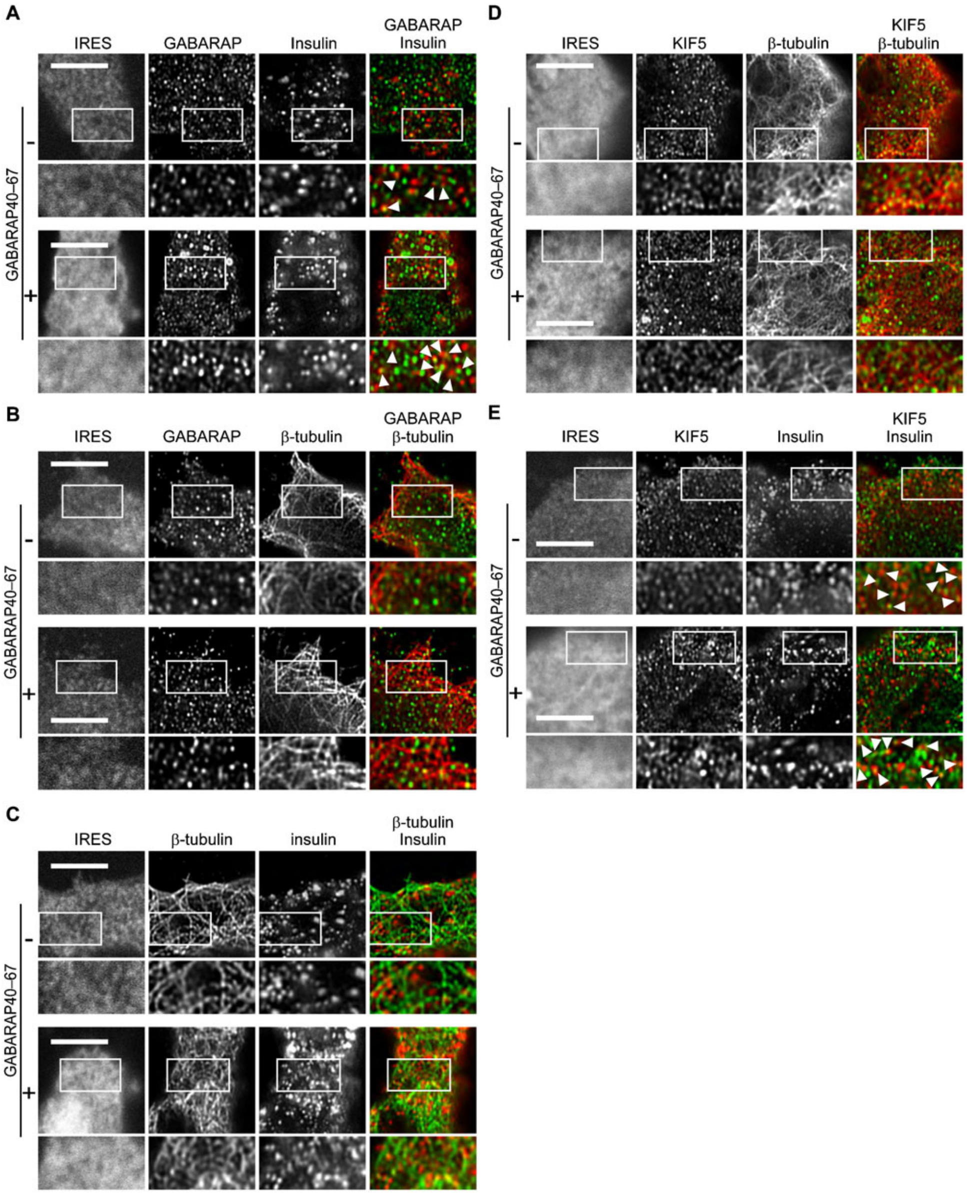

Fig. S10. Co-localization of GABARAP, insulin, β-tubulin, and KIF5 in MIN6 cells transfected with GABARAP40–67. Co-localization of GABARAP (green) and insulin (red) (A), GABARAP (green) and β-tubulin (red) (B), β-tubulin (green) and insulin (red) (C), KIF5 (green) and β-tubulin (red) (D), and KIF5 (green) and insulin (Red) (E) in MIN6 cells transfected with a pIRES2-DsRed/empty vector (upper 2 panels) and pIRES2-DsRed/GABARAP40–67 (lower 2 panels). After a 10-min exposure to 30 mM glucose, cells were fixed with 3.7% paraformaldehyde, subjected to immunocytochemistry with each specific antibody, and analyzed by confocal microscopy. Endogenous GABARAP was stained using an anti-GABARAP antibody, which recognized amino acid residues 1–39 of GABARAP. The obtained blue images were replaced with red in the merged panels. The framed area is magnified (the lower panels in each). Arrowheads indicate the colocalization of GABARAP with insulin (A) and KIF5 with insulin (E). A set of typical images from more than 40 cells in 3 independent experiments is shown. Scale bars: 5 μm.
